# Quantification of risk factors for postherpetic neuralgia in herpes zoster patients: A cohort study

**DOI:** 10.1212/WNL.0000000000002808

**Published:** 2016-07-05

**Authors:** Harriet J. Forbes, Krishnan Bhaskaran, Sara L. Thomas, Liam Smeeth, Tim Clayton, Kathryn Mansfield, Caroline Minassian, Sinéad M. Langan

**Affiliations:** From the Faculty of Epidemiology & Population Health, London School of Hygiene and Tropical Medicine, London, UK.

## Abstract

**Objective:**

To investigate risk factors for postherpetic neuralgia, the neuropathic pain that commonly follows herpes zoster.

**Methods:**

Using primary care data from the Clinical Practice Research Datalink, we fitted multivariable logistic regression models to investigate potential risk factors for postherpetic neuralgia (defined as pain ≥90 days after zoster, based on diagnostic or prescription codes), including demographic characteristics, comorbidities, and characteristics of the acute zoster episode. We also assessed whether the effects were modified by antiviral use.

**Results:**

Of 119,413 zoster patients, 6,956 (5.8%) developed postherpetic neuralgia. Postherpetic neuralgia risk rose steeply with age, most sharply between 50 and 79 years (adjusted odds ratio [OR] for a 10-year increase, 1.70, 99% confidence interval 1.63–1.78). Postherpetic neuralgia risk was higher in women (6.3% vs 5.1% in men: OR 1.19, 1.10–1.27) and those with severely immunosuppressive conditions, including leukemia (13.7%: 2.07, 1.08–3.96) and lymphoma (12.7%: 2.45, 1.53–3.92); autoimmune conditions, including rheumatoid arthritis (9.1%: 1.20, 0.99–1.46); and other comorbidities, including asthma and diabetes. Current and ex-smokers, as well as underweight and obese individuals, were at increased risk of postherpetic neuralgia. Antiviral use was not associated with postherpetic neuralgia (OR 1.04, 0.97–1.11). However, the increased risk associated with severe immunosuppression appeared less pronounced in patients given antivirals.

**Conclusions:**

Postherpetic neuralgia risk was increased for a number of patient characteristics and comorbidities, notably with age and among those with severe immunosuppression. As zoster vaccination is contraindicated for patients with severe immunosuppression, strategies to prevent zoster in these patients, which could include the new subunit zoster vaccine, are an increasing priority.

Postherpetic neuralgia (PHN) is the commonest complication of herpes zoster^1^ and may cause severe pain.^2^ The lifetime incidence of zoster is 30% and an estimated 12.5% of zoster patients aged ≥50 years develop PHN.^3^ Symptoms can persist for months or years, often profoundly affecting a patient's quality of life.^4^ There are no known effective disease-modifying therapies for PHN. Treatments target symptom control, yet are often inadequate at relieving pain and are ineffective in almost half of patients with PHN.^5–7^

To date, observational and trial data have provided inconsistent evidence that administering antivirals at rash onset reduces PHN risk.^8^ These trials also tend to exclude immunosuppressed patients, such that the efficacy of antivirals to prevent PHN in this patient group is greatly under-researched. The only proven and available intervention to reduce PHN risk is through varicella-zoster virus vaccination.^9^ However, the high cost of the vaccine means many countries limit its coverage. A recent review demonstrates our incomplete understanding of PHN risk factors^10,11^; except for age, evidence is conflicting and studies are often underpowered to detect associations. Considering the dearth of effective treatment options for PHN, identifying PHN risk factors to inform zoster vaccination policy could have important public health benefits.^12^

This article aims to quantify risk factors for PHN in a large prospective study among zoster patients. It also investigates whether antivirals modify the effect of these risk factors on PHN.

## METHODS

### Study design and setting.

We conducted a study among zoster patients using prospectively collected data from the Clinical Practice Research Datalink (CPRD), a large routinely collected UK database of anonymized primary care records. CPRD is broadly representative of UK patient and practice characteristics.^13^ Sixty percent of CPRD patients had data available in the Hospital Episode Statistics (HES) database (version 9), a linked database of hospital attendances in England from 1997. Clinical diagnoses are coded in CPRD with Read codes and in HES with ICD-10 codes.^13^

### Study population.

The study cohort included patients with first ever zoster (identified previously^14^), followed up to determine who develops PHN. Briefly, these patients were diagnosed with zoster between January 1, 2000, and December 31, 2011, in CPRD (Read code for zoster and >12 months follow-up in CPRD before zoster diagnosis [to ensure the code represented incident zoster]) or HES (ICD-10 code for zoster in the primary diagnosis field of any episode). The zoster vaccine was introduced into the United Kingdom in 2013, therefore this was an unvaccinated population.

### Definition of PHN.

Our underlying definition of PHN was pain persisting ≥90 days following zoster diagnosis.^7^ The primary definition of PHN included patients classified as having diagnosed, probable, or possible PHN, based on a validated algorithm of PHN within a US administrative database utilizing diagnosis codes and prescription data.^15^ See table 1 for full definition.

**Table 1:**
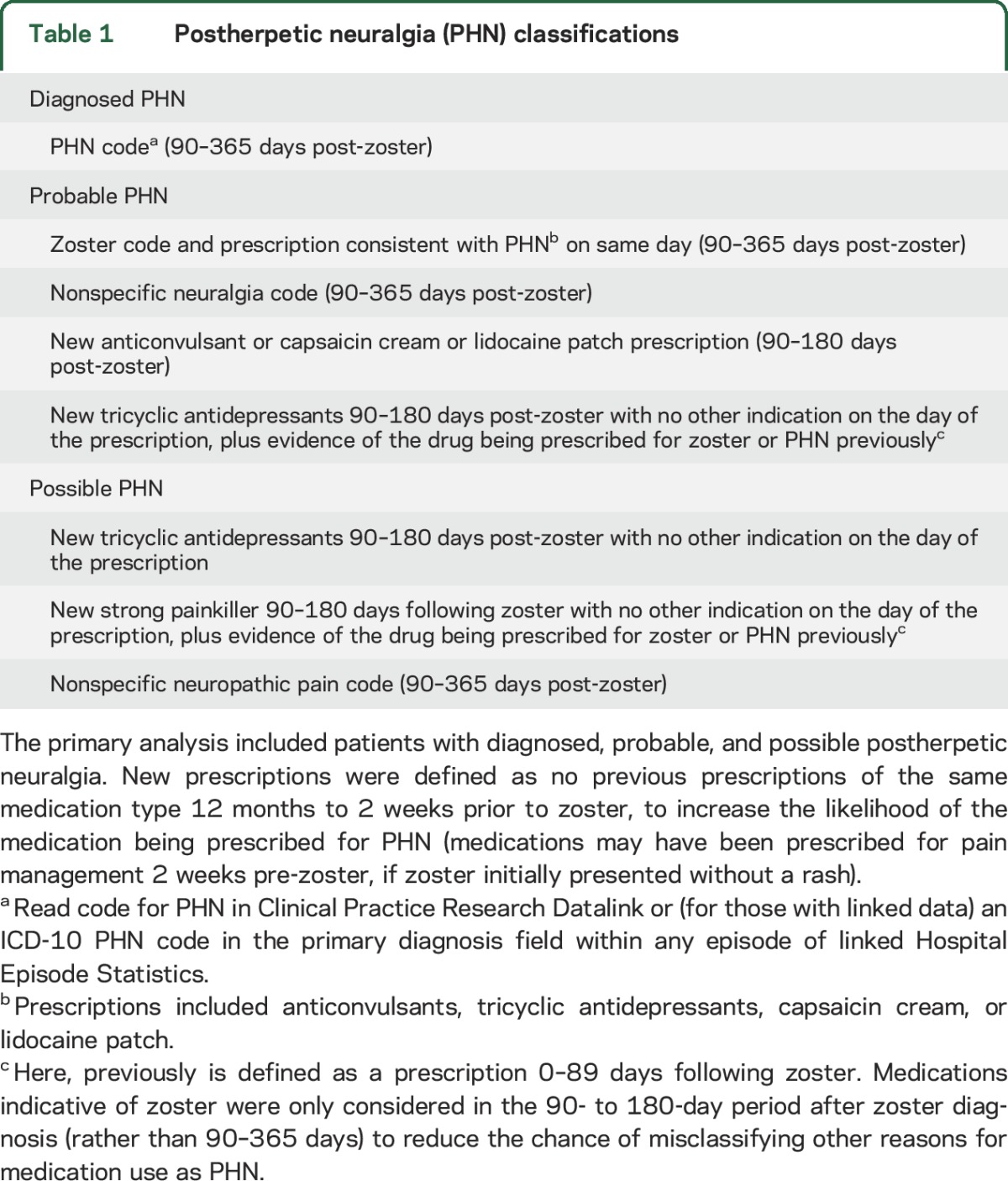
Postherpetic neuralgia (PHN) classifications

#### Exclusions.

As anticonvulsant prescriptions were part of our PHN algorithm (table 1), patients with other indications for anticonvulsants (e.g., epilepsy) recorded pre-zoster were excluded. We also excluded patients without 365 days follow-up after zoster diagnosis; we could not know whether individuals censored before 365 days without PHN met our PHN definition, which used data to 365 days.

### Risk factors of interest.

Demographic risk factors included age at zoster, sex, and socioeconomic status. Comorbidities included severely immunosuppressive conditions, specifically a recent history (<2 years before zoster diagnosis) of leukemia or lymphoma, or any history of HIV, hematopoietic stem cell transplantation, myeloma, or other unspecified cellular immune deficiencies (e.g., pancytopenia), ≥14-day course of high-dose (≥20 mg/d) oral corticosteroids, or exposure to other immunosuppressive therapies, in the month prior to zoster diagnosis. Other comorbidities included autoimmune conditions (systemic lupus erythematosus [SLE], rheumatoid arthritis [RA], inflammatory bowel disease [IBD]), diabetes, chronic obstructive pulmonary disease (COPD), asthma, chronic kidney disease (CKD), personality disorder, recent depression, and recent cancer. Other characteristics included smoking, body mass index (BMI), site of acute zoster rash, and antivirals given within 7 days of zoster diagnosis (see appendix e-I on the *Neurology*® Web site at Neurology.org for further details on risk factor definitions).

### Statistical analysis.

Crude and age-adjusted odds ratios (ORs) were calculated to estimate the strength of association of each potential risk factor with PHN at 90 days (defined above), using logistic regression. The age–PHN association was nonlinear; therefore age was modeled as a 5-knot restricted cubic spline. To numerically summarize the effect of nonlinear age on PHN risk, we also fitted piecewise linear models (see appendix e-II for further details on modeling of age).

Multivariable analyses using logistic regression were carried out on patients with complete data for all variables. All variables were included in the final model. Two models were built, first excluding then including immunosuppressive therapies, to assess whether some of the overall effects of diseases were mediated by their treatments.

Antivirals given at zoster may modify the risk of PHN. Antivirals are given to approximately 60% of patients with zoster in CPRD.^16^ We calculated stratum-specific ORs for each variable in the multivariable model by whether patients received antivirals. Patients potentially receiving antivirals in hospital were excluded from this analysis. In a post hoc analysis, we also calculated the adjusted OR for the effect of antivirals on PHN risk among patients with immunosuppression using logistic regression.

Finally, a potentially effect-modifying role of age was investigated by computing stratum-specific ORs for each variable in the multivariable model by age groups <70 years and ≥70 years as well as <60 years and ≥60 years (age groups chosen to reflect different vaccination ages, e.g., ≤60 years in the United States^17^ and 70, 78, or 79 years^18^ in the United Kingdom).

General practice was included in all models as a random effect to account for clustering, as data might be correlated within practices.

#### Sensitivity analyses and bias assessments.

We repeated the main risk factor analysis (1) for PHN at 30 days following zoster; (2) restricting PHN cases to diagnosed PHN only (see table 1 for PHN definition); and (3) excluding possible misdiagnosed herpes simplex patients (see appendix e-1 for definition of possible herpes simplex patients). We tested for health care utilization bias by assessing the association between PHN and hypothyroidism (a chronic condition requiring high-level health care use,^19^ not associated with PHN) and calculated mean yearly consultation rate pre-zoster to confirm hypothyroidism patients had the same opportunity for PHN diagnosis (i.e., via general practitioner [GP] contact). We used multiple imputation by chained equations^20^ as an alternative approach to account for missing data (9% of patients had missing data for BMI or smoking) (appendix e-III).

Bias may be introduced from excluding patients with <365 days follow-up post-zoster if those included had a different association between our risk factors and PHN, compared to those excluded. We repeated the main analysis, restricting our outcome definition to PHN identified up to 120 and 180 days after zoster; the follow-up requirements are less, thus any selection biases due to excluding patients with insufficient follow-up is reduced.

### Standard protocol approvals, registrations, and patient consents.

The study was approved by ISAC (application no. 11028) and the London School of Hygiene and Tropical Medicine (application no. 5930).

## RESULTS

Of 144,959 patients with zoster, 119,413 were eligible for inclusion (figure e-1). Almost 60% of the sample was female (table 2) and the median age was 61 years (interquartile range 48–72, range 18–101). In total, 6,956 zoster patients (5.8%) developed PHN.

**Table 2:**
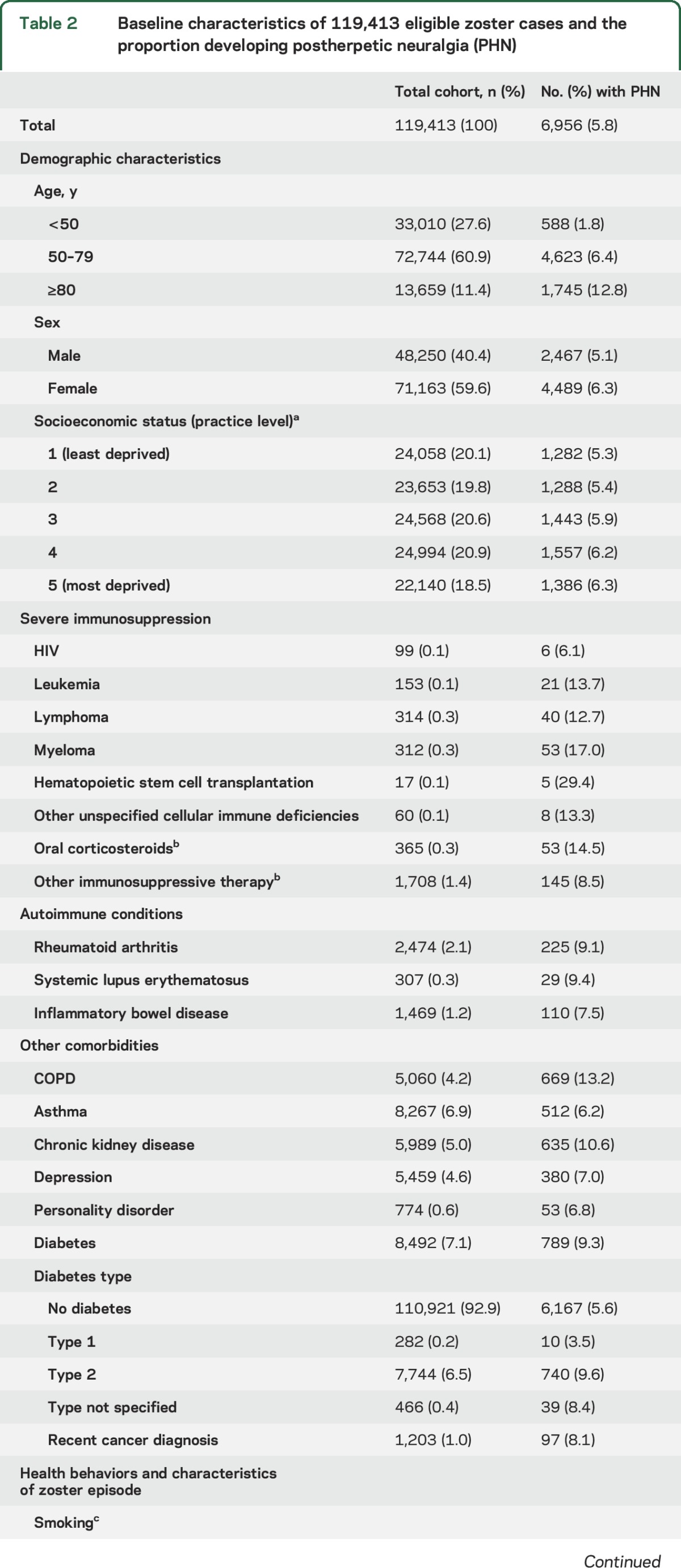

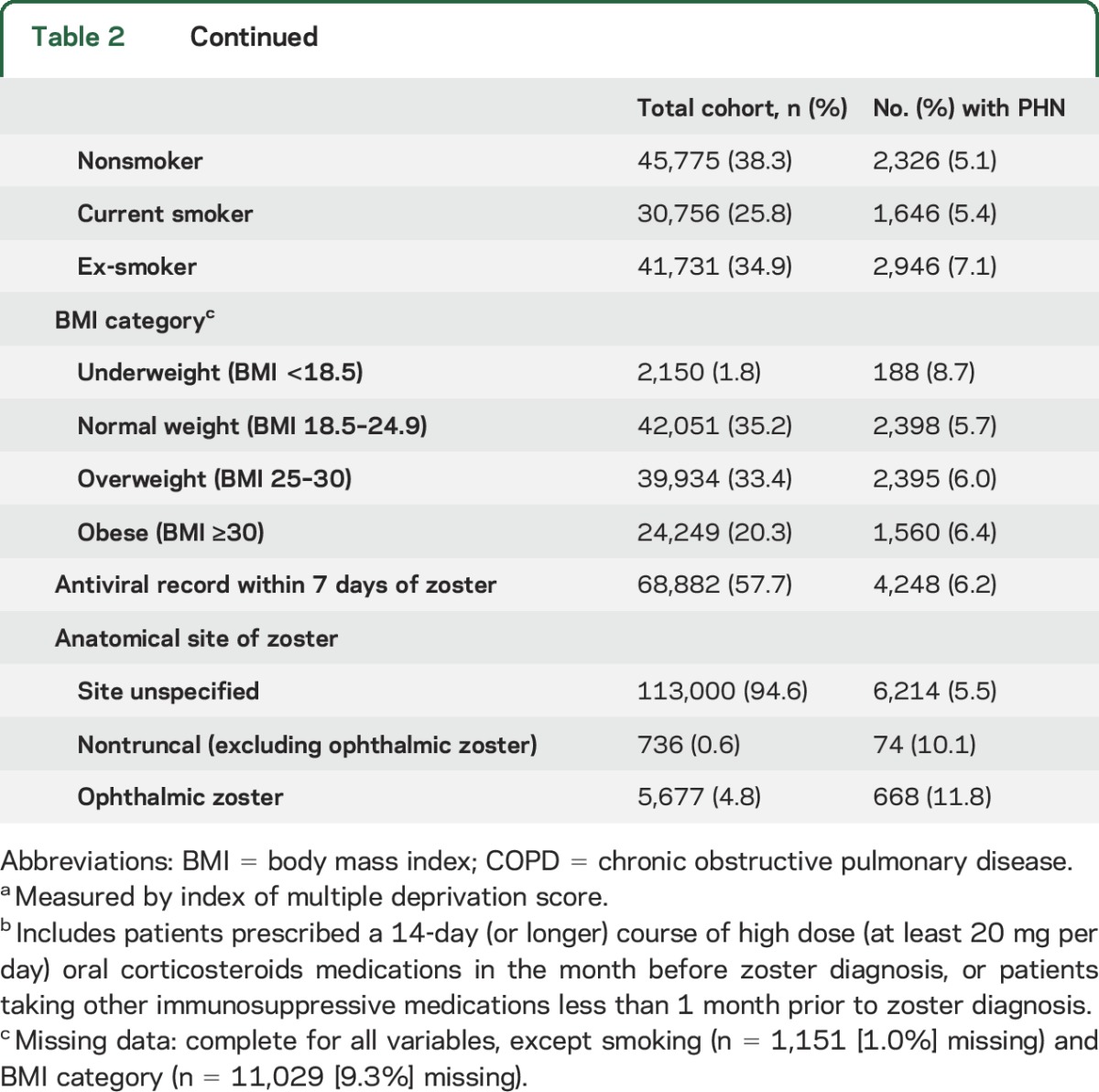
Baseline characteristics of 119,413 eligible zoster cases and the proportion developing postherpetic neuralgia (PHN)

The risk of PHN rose with age (figure e-2) and the association was nonlinear (*p* < 0.001, figure e-3). Patients 50–79 years had the steepest increased risk of PHN (table 3); a 10-year increase in age within this age band was associated with 70% increased risk of PHN (adjusted OR [adjOR] 1.70, 99% confidence interval [CI] 1.63–1.78), whereas the effect of a 10-year increase in age was attenuated above age 80 years (adjOR 1.10, 99% CI 0.94–1.28). Women were more likely to develop PHN than men, an association that remained after adjustment (adjOR 1.19, 99% CI 1.10–1.27) (table 3).

**Table 3:**
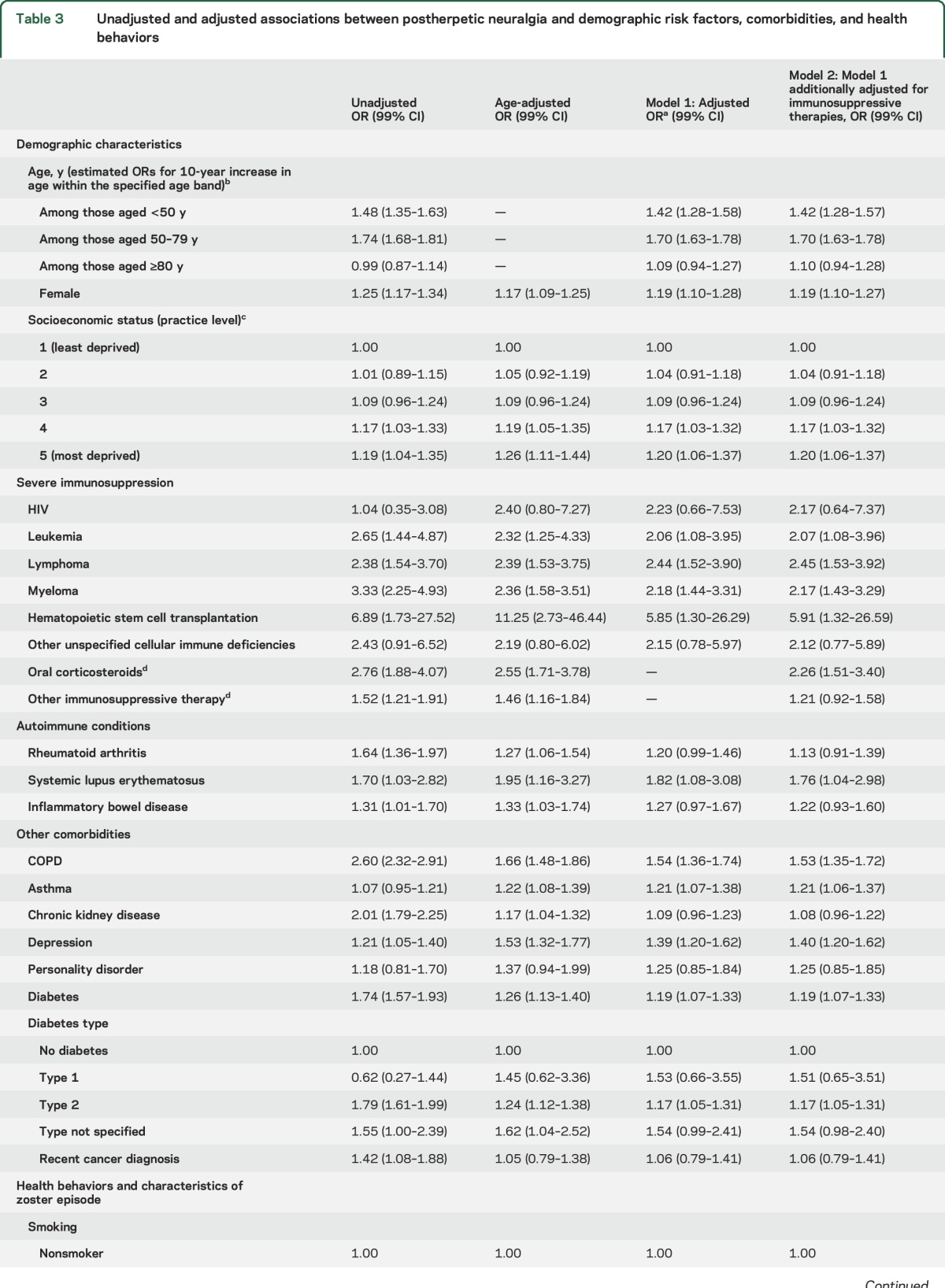

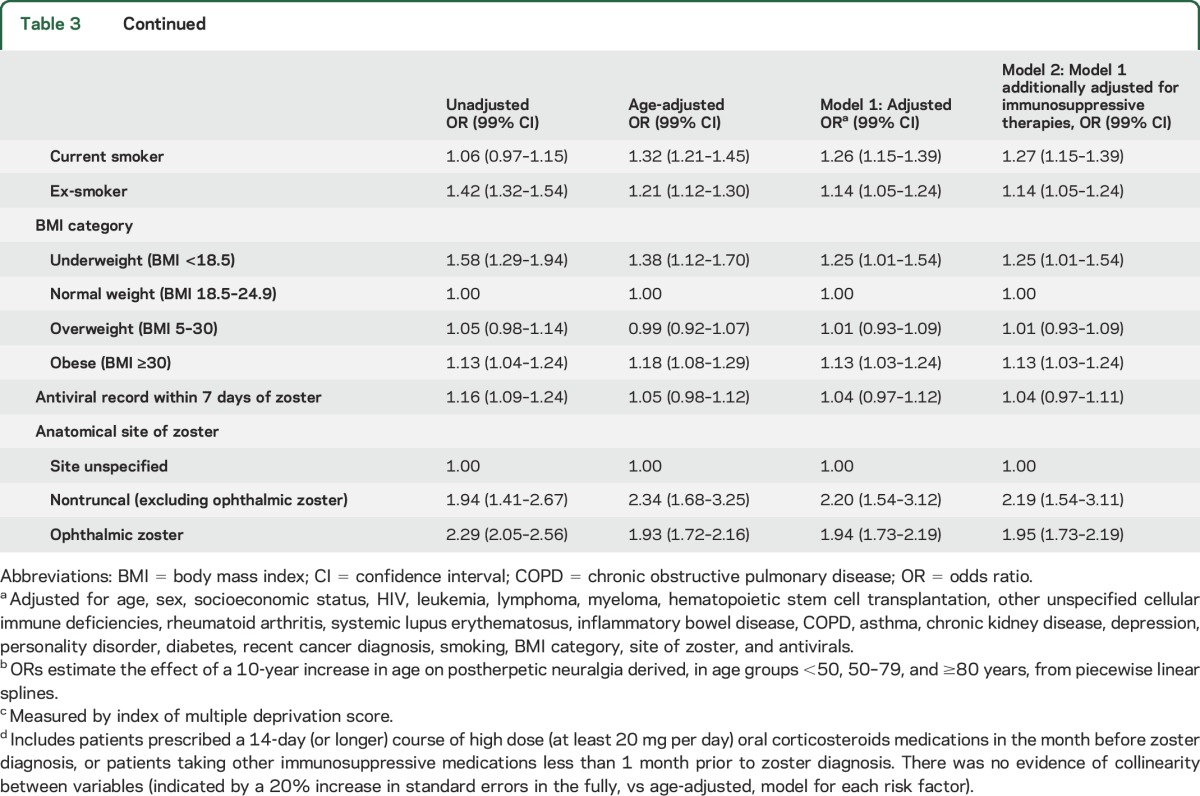
Unadjusted and adjusted associations between postherpetic neuralgia and demographic risk factors, comorbidities, and health behaviors

Conditions and therapies causing severe immunosuppression were strong risk factors for PHN (tables 2 and 3). Patients with leukemia or lymphoma in the previous 2 years, myeloma or other unspecified cellular immune deficiencies ever, or recently taking high-dose oral corticosteroids had over twice the risk of developing PHN, compared to unaffected patients. For HIV, the point estimate was also large; however, only 6 of 99 HIV patients developed PHN, hence the evidence for an association was weak (adjOR 2.17, 99% CI 0.64–7.37).

Of the autoimmune conditions, SLE was most strongly associated with PHN (adjOR 1.76, 99% CI 1.04–2.98) (tables 2 and 3). RA and IBD were also associated with a higher PHN risk (9.1% and 7.5%, respectively), with some evidence they were associated with between 10% and 20% increased risk of PHN after adjustment for confounders; the effects of these 2 conditions were less pronounced after adjusting for immunosuppressive drugs.

Of the other comorbidities assessed (tables 2 and 3), COPD was associated with a 53% increased risk (PHN risk 13.2%; adjOR 1.53, 99% CI 1.35–1.72) and recent depression a 40% increased risk (PHN risk 7.0%; adjOR 1.40, 99% CI 1.20–1.62). Asthma and type 2 diabetes were associated with 20% increased risk of PHN. After fully adjusting for confounders, personality disorder, CKD, or recent cancer diagnosis were not associated with PHN.

The overall risk of PHN among current smokers was lower than the study sample overall (5.4% vs 5.8%); however, after adjusting for age, there was around 30% increased risk of PHN in current smokers (adjOR 1.27, 99% CI 1.15–1.39) (table 2). Being underweight or obese was also associated with PHN. PHN was more prevalent in patients with nontruncal zoster (tables 2 and 3).

**Figure 1:**
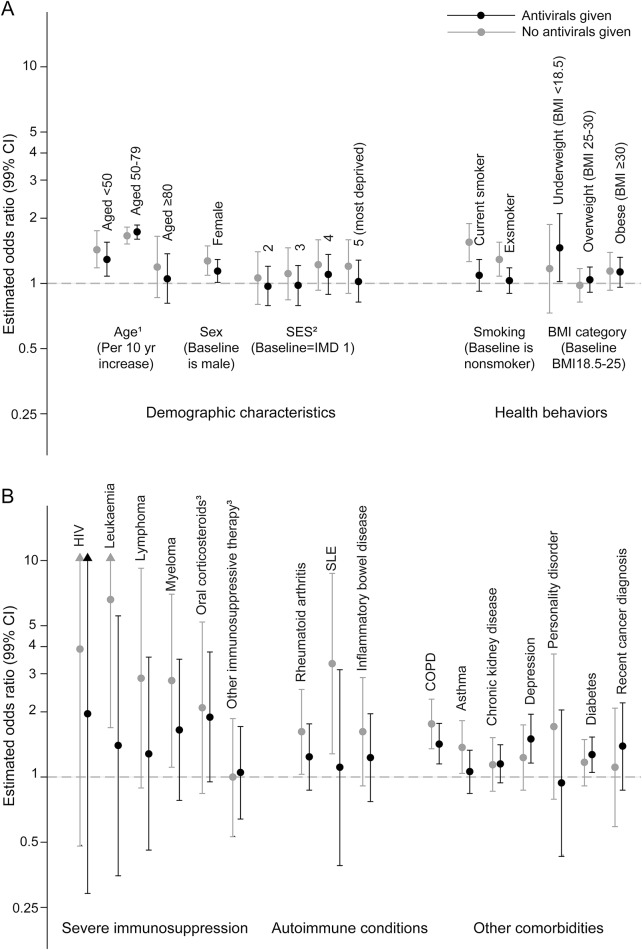
Adjusted associations between postherpetic neuralgia and demographic risk factors and health behaviors and comorbidities, stratified by whether a patient received antivirals during acute zoster Adjusted associations between postherpetic neuralgia and (A) demographic risk factors and health behaviors and (B) comorbidities, stratified by whether a patient received antivirals during acute zoster. Analyses are restricted to 69,661 patients for whom antiviral status was most likely to be available. Full results can be found in table e-8. Adjusted for age, sex, socioeconomic status (SES), HIV, leukemia, lymphoma, myeloma, immunosuppressive therapies, rheumatoid arthritis, systemic lupus erythematosus (SLE), inflammatory bowel disease, chronic obstructive pulmonary disease (COPD), asthma, chronic kidney disease, depression, personality disorder, diabetes, recent cancer diagnosis, smoking, and body mass index (BMI) category. Hematopoietic stem cell transplantation and other unspecified cellular immune deficiencies were excluded due to too few numbers. Patients excluded were those with zoster diagnosed in Hospital Episode Statistics (HES) or having a HES visit for zoster 7 days after diagnosis (n = 494), patients who were not HES-linked (n = 45,418), and patients with nontruncal zoster (n = 3,840), as their antiviral use may not be recorded in Clinical Practice Research Datalink. ^1^Odds ratios estimate the effect of a 10-year increase in age on postherpetic neuralgia derived, in age groups <50, 50–79, and ≥80 years, from piecewise linear splines. ^2^Measured by index of multiple deprivation score (IMD1 = least deprived, IMD5 = most deprived). ^3^Includes patients prescribed a 14-day (or longer) course of high dose (at least 20 mg per day) oral corticosteroids medications in the month before zoster diagnosis, or patients taking other immunosuppressive medications less than 1 month prior to zoster diagnosis. Interaction terms between antiviral use and other risk factors were added to the model one at a time. CI = confidence interval.

The effects of the risk factors on PHN were broadly similar between those given and not given antivirals, except for individuals with leukemia (*p* value for interaction 0.045), SLE (0.026), COPD (0.043), and smoking (0.002); point estimates for their effect on PHN appeared higher in those not given antivirals (figure 1). Post hoc analysis found no evidence that antivirals reduced the risk of PHN in patients with immunosuppression (appendix e-V).

The effect of COPD (*p* value for interaction 0.006), diabetes (0.004), currently smoking (0.006), or being obese (0.037) were slightly stronger in patients <70 years (table e-1); the effect of asthma (0.068), CKD (0.018), diabetes (0.018), and being underweight or obese were slightly stronger in patients <60 years (0.031) (table e-2). However, no clear patterns were observed.

### Sensitivity analyses.

We reran the main analysis varying the PHN definitions. Most CIs included the point estimate from the main analysis (table e-3), except PHN at 30 days was associated with antiviral use (adjOR 1.12, 99% CI 1.06–1.18), yet less strongly associated with ophthalmic zoster (adjOR 1.72, 99% CI 1.56–1.91); restricting the outcome to diagnosed PHN only, the effect of ophthalmic zoster (adjOR 2.67, 99% CI 2.24–3.19) and a 10-year increase in age between 50 and 79 years (adjOR 2.08, 99% CI 1.92–2.24) became stronger and the effect of female sex disappeared (adjOR 1.00, 99% CI 0.89–1.14) (table e-3).

We explored in post hoc analysis this loss of an association with female sex (using the primary PHN definition) (tables e-4 and e-5). The effect of female sex on PHN was driven by cases of PHN defined using tricyclic antidepressant use, yet we found evidence suggesting prescribing practices for PHN differ by sex (appendix e-IV).

Of 7,416 patients with hypothyroidism, 599 (8.1%) developed PHN. The fully adjusted OR between hypothyroidism and PHN indicated no association (adjOR 1.01, 99% CI 0.90–1.14, adjusted for variables in table 3, model 2); furthermore, mean yearly consultation rates among patients with our risk factors of interest were variable (table e-6), showing no consistent pattern with PHN risk.

Accounting for missing BMI and smoking data using multiple imputation made no difference in the results (table e-7). Restricting to shorter follow-up periods (specifically 120 and 180 days) also had no major effect on the results (figure e-4).

## DISCUSSION

This study shows that older age and severe immunosuppression, such as recently having lymphoma or leukemia, are the strongest risk factors for PHN among zoster patients. Although immunosuppressed patients are not currently eligible for vaccination, promising research on a new subunit zoster vaccine (HZ/su) may enable vaccination within this group.^21^ Other risk factors included autoimmune conditions (RA, SLE, and IBD), COPD, depression, diabetes, asthma, lower socioeconomic status, smoking, being underweight or overweight, and nontruncal zoster. Antivirals given at zoster were not associated with PHN risk overall, but there was some weak evidence that their use mitigated the increased risks associated with leukemia, SLE, COPD, and smoking.

This study addresses an absence in the literature of well-powered studies assessing PHN risk factors.^10,11^ The study benefits from being the largest study addressing this question; being population-based; using a dataset broadly representative of the United Kingdom^13^; tightly accounting for confounding by age; and using a more precise definition of PHN than earlier studies using routinely collected data.

Despite its strengths, this study has some limitations. Although we attempted to reduce misclassification of PHN by basing our definition on a validated method for administrative data,^15^ PHN incidence is lower than in previous studies (5.8% vs 7.2% in an Icelandic study with active follow-up of similar aged zoster cases^22^). The reported prevalence of PHN varies hugely across studies. We used a 90-day (rather than 30-day) definition and included patients <50 years old, which may explain the relatively low PHN prevalence. However, some PHN diagnoses could have been missed. Unidentified cases are likely those patients with mild pain, who used over-the-counter medications for initial pain relief or (if GP-prescribed treatments were ineffective) for follow-on pain relief. These findings are therefore likely to be generalizable to patients with severe PHN. Another explanation for the low prevalence may be that some immunocompromised patients are treated exclusively in secondary care; however, this is likely to be a few cases only, because PHN is largely treated in primary care.

Certain clinical features of acute zoster are accepted PHN risk factors, including prodromal pain and increased rash severity.^10,11^ In CPRD, these data are unavailable. These features may lie on the causal pathway or be mediators between our exposures and PHN. For example, patients with leukemia may experience greater viral load, which manifests as a severe rash and increase PHN risk. It would be inappropriate to control for rash severity, as we want the total effect of leukemia on PHN; lacking data on these clinical features is therefore unlikely to limit our findings.

In numerous studies, increasing age is associated with a sharp increase in PHN risk.^3,23^ Our study identified a nonlinear age effect; PHN risk increased steeply between 50 and 79 years and attenuated after age 80. A Dutch cohort study among 837 zoster patients^24^ suggested PHN incidence continues to increase in individuals >80 years; however, PHN was defined as analgesic prescription 3 months post-zoster, potentially causing misclassification, particularly among older individuals. Other studies report PHN risk lessening around 80 years.^25–27^ Our observed effect may arise from underascertainment of PHN in patients >80 years due to frailty preventing GP attendance or PHN-associated pain being superseded by other comorbidities.

Previous studies have not identified depression as a risk factor for PHN,^28–31^ unlike our study. We acknowledge that our result may be driven by patients requiring tricyclic antidepressants for depression, and being misclassified as PHN cases; when restricting PHN cases to diagnosed PHN only, the association with depression disappeared (adjOR 1.12, 99% CI 0.84–1.49). However, the wide CIs suggest there may have been insufficient power to detect an effect.

The study found no evidence that antivirals protected against PHN. This is unlikely to be attributed to inadequate dosing, as 93% of treated patients received at least the recommended minimum antiviral dose. However, other limitations could explain this null finding. In primary care, patients with severe zoster are recommended to receive antivirals, yet are also more likely to develop PHN. This may introduce confounding by indication and mask a protective effect. Also, trial data has assessed the efficacy of antivirals administered within 72 hours of rash onset.^8^ Although 97.5% of patients were prescribed antivirals on the day of zoster diagnosis, zoster treatment initiation may occur >72 hours after rash onset, when the effect of antivirals is unproven. Although data on time from actual rash onset to presentation at general practitioners are not available in this dataset, 2 previous UK studies suggest 50%–65% of patients present within 72 hours,^32,33^ leaving a number of patients treated after 72 hours from rash onset.

Our recent systematic review of PHN risk factors concluded there was no consensus regarding immunosuppression as a risk factor for PHN.^10^ The present study demonstrates that immunosuppression is associated with greater PHN risk and the study had sufficient power to assess the effect of specific immunosuppressive conditions and therapies on PHN risk. Our study is also novel in identifying autoimmune conditions as PHN risk factors; autoimmune conditions had scarcely been assessed previously, though SLE was associated with over 2-fold increased risk of PHN in a large Taiwanese cohort study.^34^

Disentangling the role of diseases and their drug treatments on the PHN risk is challenging. There was little evidence that the increased risks associated with severely immunosuppressive conditions were mediated by immunosuppressive drugs. However, treatment such as chemotherapy, given in secondary care, is not captured in CPRD and probably explains at least part of the increased risk. The effects of IBD and RA on PHN were less pronounced after adjusting for immunosuppressive treatments, suggesting that the association is driven predominantly by exposure to these drugs. By contrast, the effect of SLE did not appear to be mediated by immunosuppressive therapies, although CRPD lacks reliable data on newer biological therapies.

Establishing the role of severe immunosuppression and autoimmune conditions on PHN risk may help shed light on the underlying pathophysiologic mechanisms that lead to PHN, which are poorly understood. Two key etiologic hypotheses are that PHN is caused by a persistence of varicella-zoster virus following zoster or by increased neuronal excitability and alteration of pain perception.^6,35^ Our finding of a strong association between immunosuppression and PHN risk seems to support the former hypothesis; lower cell-mediated immunity may lead to higher levels of virus during acute infection and thus an increased risk of PHN.

Cost-effectiveness studies are needed to determine the value in vaccinating patients with identified risk factors for PHN against zoster. Those of older age and with severe immunosuppression were at the highest risk of PHN. Strategies to prevent zoster in patients with severe immunosuppression are an increasing priority as these patients are not currently eligible for zoster vaccination, although the new development of a subunit vaccine provides a promising alternative.^21^

## Supplementary Material

Data Supplement

## GLOSSARY

adjOR: adjusted odds ratio
BMI: body mass index
CI: confidence interval
CKD: chronic kidney disease
COPD: chronic obstructive pulmonary disease
CPRD: Clinical Practice Research Datalink
GP: general practitioner
HES: Hospital Episode Statistics
IBD: inflammatory bowel disease
ICD-10: International Classification of Diseases–10
OR: odds ratio
PHN: postherpetic neuralgia
RA: rheumatoid arthritis
SLE: systemic lupus erythematosus
