# Supplementary material for: Quantification of risk factors for postherpetic neuralgia in herpes zoster patients: A cohort study
Source: Neurology. 2016 Jul 5;87(1):94–102. doi: 10.1212/WNL.0000000000002808 (PMC4932239; doi:10.1212/WNL.0000000000002808)
Supplement: Data Supplement [file supp_87_1_94__index.html]

Quantification of risk factors for postherpetic neuralgia in herpes zoster patients — Data Supplement 

# Quantification of risk factors for postherpetic neuralgia in herpes zoster patients

## Data Supplement

**Neurology® data supplements are not copyedited before publication. Published editorials and translations have been copyedited.  
 © 2016 American Academy of Neurology.  
  
 Files in this Data Supplement:**

- Appendix e-1 - Microsoft Word file
