# Supplementary material for: Quantification of risk factors for postherpetic neuralgia in herpes zoster patients: A cohort study
Source: Neurology. 2016 Jul 5;87(1):94–102. doi: 10.1212/WNL.0000000000002808 (PMC4932239; doi:10.1212/WNL.0000000000002808)
Supplement: Data Supplement [file supp_WNL.0000000000002808_Appendix_e-1.docx]

**APPENDIX**

**e-1: Further details on definitions of risk factors**

***Severe Immunosuppression***

Our models included severely immunosuppressive conditions determined to be vaccine contraindications by the Advisory Committee on Immunization Practices,[^1^](#_ENREF_1) namely recent history (<2 years before zoster diagnosis) of leukaemia or lymphoma, or any history of HIV, hematopoietic stem cell transplantation, myeloma or ‘other unspecified cellular immune deficiencies’ (e.g. pancytopenia). Use of immunosuppressive therapy was also included; all relevant prescriptions prior to zoster diagnosis were extracted, prescription duration was calculated (using data on quantity of tablets prescribed and numeric daily dose). Oral corticosteroid exposure was defined as a 14-day course of high-dose (≥20 mg/day) oral corticosteroids in the month prior to zoster diagnosis. Exposure to other immunosuppressive therapies in the month prior to the zoster diagnosis was included as an additional covariate.

***Other comorbidities***

We assessed three autoimmune conditions; *rheumatoid arthritis, systemic lupus erythematosis and inflammatory bowel disease.* These were defined as a diagnosis prior to the zoster diagnosis.

*Chronic obstructive pulmonary disorder* patients were defined as those with a diagnosis of chronic obstructive pulmonary disorder, including chronic bronchitis and emphysema, prior to the zoster diagnosis and ≥35 years at first chronic obstructive pulmonary disorder diagnosis).

*Asthma* patients were those with an asthma diagnosis before the zoster diagnosis and an asthma-related prescription [short and long-acting beta-2 agonists and antimuscarinics, inhaled corticosteroids, cromoglycates and nedocromil, theophyllines, leukotriene receptor agonists and omalizumab] within 12 months prior to the zoster diagnosis; patients with a chronic obstructive pulmonary disorder diagnosis ever in their medical history were not classified as asthmatic.

*Chronic kidney disease* patients were those with a diagnosis of mild, moderate or severe chronic kidney disease, kidney transplant or kidney dialysis any time prior to the zoster diagnosis.

*Depression* was defined as having a diagnosis or symptom of depression (such as “feeling depressed” or “sad mood”) within one year prior to the zoster diagnosis; symptoms were included due to the trend post-2004 of using symptom rather than diagnosis codes in UK primary care.[^2^](#_ENREF_2)

To define *diabetes* we required a definite diabetes diagnosis, or a possible diabetes code [e.g. self- monitoring of blood glucose] with a subsequent diabetes-specific prescription [insulin or oral anti-diabetics], or ≥2 diabetes drug prescriptions prior to the zoster diagnosis; gestational diabetes and drug-induced diabetes were excluded. We also used age at first diagnosis, age at first treatment and treatment received to classify patients into Type 1 or 2 diabetes. Distinguishing between type 1 and type 2 diabetes is not always possible from diabetes codes as patients are frequently given a non-specific code. Furthermore, where type of diabetes is assigned, it has been found to be unreliable.[^3^](#_ENREF_3) Therefore we chose not to use this information, but instead use age at first diagnosis, age at first treatment and treatment received to classify diabetes type, as in previous Clinical Practice Research Datalink studies.[^4^](#_ENREF_4) [^5^](#_ENREF_5) Type 1 was assigned where; age at first diagnosis was ≤35 years and treatment ever was exclusively insulin, or patients received at least two insulin prescriptions ≤35 years, but had no diabetes diagnosis. Type 2 was assigned where; age at first diabetes diagnosis was >35; or patients received exclusively oral anti-diabetics’s >35 years. Patients with age at diagnoses >35 but treated exclusively with insulin and those not fitting into these categories were assigned as “Type not specified”.

*Recent cancer* was defined as *any* cancer diagnosed in previous year (excluding leukaemia and lymphoma previously categorised as severely immunosuppressive).

***Health behaviours features of acute zoster***

For *smoking*, data were derived from medical Read codes and data from the additional details file. For *BMI*, only data from the additional details files was used, as Read codes classifying patients by BMI category are very rarely recorded. Where patients had multiple recordings, the nearest status in the period -1y to +1month from zoster diagnosis date was taken (best); if not available, then the nearest in the period +1month to +1y after zoster diagnosis date was taken (second best); if not available, then the nearest before -1y from zoster diagnosis date was taken (third best); if not available, then take nearest after +1y from zoster diagnosis date was taken (least best).

*Site of acute zoster* was identified from the presence of specific zoster diagnostic codes. Some Read and ICD codes specify the site of zoster (such as, “Herpes zoster with dermatitis of eyelid”); codes indicating site within 12 months after first diagnosis were used. In line with a previous study identifying ophthalmic zoster in CPRD, we also defined ophthalmic zoster as patients with nonspecific zoster, plus a diagnosis of, or treatment for, acute eye infection (such as keratitis or conjunctivitis) within 2 weeks of zoster onset or from records of first-ever specific chronic eye conditions known to be associated with zoster (such as, conjunctival scarring or episcleritis), within 3 months after zoster onset.[^6^](#_ENREF_6)

*Antiviral use at acute zoster* was identified through prescription of acyclovir, valaciclovir or famciclovir within 7 days from zoster diagnosis.

***Possible misdiagnosis of herpes simplex as herpes zoster***

Of the 119,413 cohort, 1586 (1.6%) patients had a further zoster code 90-365 days after their first zoster. Of these 215 (13.6%) had a PHN medication or a PHN diagnostic code, and in the main analysis these were categorised as PHN. However, the other 1371 patients had a zoster code without medications or codes suggesting PHN. There were three possible explanations for these patients. The first is that they were recurrent zoster; however this is very rare in immunocompetent patients, who made up 96% of the group. The second is that these are poorly coded PHN patients, however it seems unlikely that no PHN prescriptions would be given. The third, and perhaps most plausible explanation is that these patients were misdiagnosed herpes simplex cases. Herpes simplex is known to recur more frequently than zoster and can, albeit rarely, present with dermatomal distribution similar to herpes zoster. [^7^](#_ENREF_7) Further to this, half of the patients were prescribed an antiviral at their later zoster diagnosis, which may further indicate misdiagnosis of herpes simplex. Therefore as a sensitivity analysis we excluded all 1586 patients with a further zoster code following first zoster diagnosis.

Risk factors we are unable to assess included: physical trauma[^8^](#_ENREF_8) or surgical intervention at site of zoster,[^8^](#_ENREF_8) genetic factors, ethnicity and functional status[^9^](#_ENREF_9) (appendix section A-I for more details on these variables).

**e-II: Modelling age**

1. Using restricted cubic splines to adjust for age

There was very good evidence that the relationship between PHN and age at zoster diagnosis was not linear (test for departure from linear trend P<0.001); in other words there was no evidence of a constant increase in the log odds of PHN per unit increase in age.

Categorizing age into groups was considered but not pursued as this may lead to loss of information. Splitting age into categories would assume there is a single exposure effect within that category; considering age is such a strong risk factor for PHN this may be an unrealistic assumption. Further to that, cut-points are often arbitrarily chosen potentially heavily influencing the apparent shape of the age-PHN relationship.[^10^](#_ENREF_10) Allowing for a non-linear relationship would improve our understanding of the relationship between age and PHN, reduce the possibility of residual confounding by age when including it as a confounder and improve the model from a statistical viewpoint.

We therefore utilised restricted cubic splines to make better use of within category information and allow for non-linearity. Splines are a type of smoothing function which provide a continuous curve rather than a step function. Cubic splines are one type of spline where the effect measure is regressed on a cubic function of exposure (here, age), across several different regions or categories of exposure and spanning the entire range of exposure. So cubic splines consist of piecewise cubic polynomials (in other words curves with up to 2 turning points) between specified “knots”. The “restricted” means the relationships will be linear before the first knot and after the last knot. A single smooth curve across these regions is then produced.

As there was no obvious biological rationale for where to place the knots, they were placed at equal percentiles of age. The number of knots was chosen by assessing the Akaike information criterion (AIC) when 3 to 7 knots were included: the AIC criterion is a measure of the relative quality of a model given a set of data and thus provides a method to select the most appropriate model. AIC rewards goodness of fit of a model but includes a penalty for increasing number of parameters; this therefore discourages over-fitting (including more parameters will almost always improve the fit of the model). The model with the minimum AIC is preferred. Here, the AIC reduced from 49825 to 49758 when modelling it as a 3-knot and 5-knot respectively. The reduction in AIC beyond 5 knots was marginal (6 knots: 49753, 7 knots: 49728), therefore 5 knots were chosen (with knots at 26, 49, 61, 71, 84 years).

1. Using piecewise linear splines to estimate the effect of age on PHN

Restricted cubic splines provide a smooth curve of the age-PHN relationship and impose few constraints on that curve; however, they have the drawback of not providing interpretable parameters. Linear splines were used to estimate the relationship between age and PHN as a piecewise linear function, in other words a function composed of linear (straight line) segments between the specified knots. The points (knots) at which the linear segments join were determined by first, looking at the restricted cubic spline graph to assess where the effect of age on PHN appeared to alter. We then fitted piecewise linear models across various possible threshold values and used the one giving the lowest AIC. The knots were at 50 and 80 years.

**e-III: Dealing with missing data**

We used multiple imputation to account for missing data. Missing data was present for BMI and smoking. In total, 91% percent of patients had complete data for all variables. To maximise the use of the data while properly incorporating the extra uncertainty arising due to missing data, multiple imputation by chained equations[^11^](#_ENREF_11) was used to impute missing values for BMI and smoking from multinomial models. The imputation model included all covariates from the main outcome model (Model 2 in table 2), with age included as a 5-knot restricted cubic spline. We also included extra comorbidities, identified using medical Read codes, to look for additional markers of BMI or smoking related diseases. These included: stroke, peripheral artery disease, angina (stable and unstable), acute coronary syndrome, congestive heart failure, myocardial infarction, hypertension and alcoholic liver disease (including portal hypertension) and pancreatitis. Five imputed datasets were created and combined for analysis. Distributions of imputed values were visually checked for comparability with the observed data. This was not done as the primary approach due to possible violation of the “missing at random” assumption for BMI and smoking.

**e-IV: Investigating gender as a risk factor for PHN**

In a sensitivity analysis which restricted the PHN definition to diagnosed PHN only (that is, patients with a Read code for PHN 90-365 days following zoster), female gender was no longer associated with an increased risk of PHN (table e-2, sensitivity analysis 2). We carried out some post-hoc analysis to investigate this further.

*Gender distribution by PHN classification (diagnosed, probable and possible PHN)*

The proportion of females among those with diagnosed, probable and possible PHN was 61.8%, 65.4%, 66.4% respectively. The effects of gender on PHN risk showed some variation across PHN classifications (see Table e-3); gender was not associated with diagnosed PHN, whilst females appeared to be at increased risk of probable and possible PHN. The association between gender and PHN according to the exact source of evidence for PHN was calculated (see table e-6); PHN defined from prescription of tricyclic antidepressants was driving the increased risk of probable and possible PHN in females (see table e-4, analysis A).

Three hypotheses were suggested and explored to explain this pattern:

1. *Patients with depression were being misclassified as PHN cases:* Of particular concern was that PHN cases defined from tricyclic antidepressant use were actually patients with depression, a condition widely known to be more common in females. To explore whether there may be systematic misclassification of PHN, the effects of gender on PHN risk were stratified by history of mental health problems ever in CPRD prior to zoster. These mental health problems included symptoms or diagnoses suggesting depression, anxiety, bipolar, or suicidal ideation. In both strata there appeared to be an increased risk of tricyclic antidepressant-defined PHN, suggesting the association between PHN and gender isn’t driven entirely by previous mental health problems (see table e-4, analysis B and C). As a secondary check, patients with depression (using depression diagnosis and depression symptom codes) in the year prior to zoster or the year following zoster were excluded; there was still an increased risk of PHN defined by tricyclic antidepressant use among females (see table e-6; analysis D).
2. *Females with PHN are more likely to be prescribed medications than males with PHN:* Among 2156 patients with “diagnosed PHN”, 33% (701/2156) did not receive any PHN medications 90-180 days following zoster. It was hypothesised that females may be more likely to receive treatment when visiting their GP; however, there was little difference in the proportion of male and females prescribed medications 90-180 days following zoster (69.8% vs 66.1% respectively, chi-squared P-value=0.07).
3. *Females with PHN are more likely to receive a tricyclic antidepressant than males with PHN:* Among patients with diagnosed PHN who received treatment 90-180 days following zoster (n=1455), females were more likely to receive tricyclic antidepressants in the 90-180 day period than males; 43.6% (384/880) females received a tricyclic, compared to 34.8% (200/575) of males (chi² P-Value=0.001). This suggests GPs may have different prescribing practices for males and females, which may explain why PHN defined through tricyclic antidepressant use is associated with gender.

We therefore concluded that the association between gender and probable and possible PHN reflects different prescribing practices; specifically that female PHN patients are more likely to be given a tricyclic antidepressant compared to male PHN patients.

**e-V: Effect of antivirals in patients with immunosuppression**

*Post-hoc* analysis explored whether antivirals reduced the risk of PHN in patients with immunosuppression. Among patients with *any* severely immunosuppressive condition (grouped due to small numbers; n=1614), 8.3% of those given antivirals developed PHN (86/1,043), compared to 10.9% not given antivirals (62/571); however this was compatible with chance variation (OR for the effect antiviral use on PHN risk, among those with severe immunosuppression: 0.73, 99%CI:0.45-1.18, adjusted for variables in Table 2 Model 2).

Using antivirals to prevent PHN in patients with immunosuppression is under-researched; many trials exclude these patients.[^12^](#_ENREF_12) In this *post-hoc* analysis, the rate of PHN among severely immunosuppressed patients given antivirals was lower, than those not given antivirals, but the confidence interval around the OR was wide and included one, reflecting the small numbers available. Given that *a priori* we assumed antiviral use would be associated with greater risk of PHN (due to confounding by indication; patients with severe zoster are more likely to develop PHN and receive antivirals) a lower risk of PHN in this group, albeit non-significant, suggests antiviral effectiveness in patients with severe immunosuppression should be investigated further. Capture of antiviral therapy among the more severely immunosuppressed patients may be poor, as patients could be immediately referred for intravenous antiviral therapy; therefore differential misclassification of antiviral exposure may be driving the higher risk of PHN in the “no antivirals” group.

**Figure e-1:** Flow diagram describing the identification of diagnosed, probable and possible postherpetic neuralgia

Patients with first ever zoster in CPRD or HES: N=144,959

Not PHN:

N=112,457

NO

NO

NO

NO

NO

NO

NO

NO

Probable PHN:

N=3007

Notes

TCA: tricyclic antidepressants. **NEW** medications defined as no previous prescription of the same drug class 12 months to two weeks prior to zoster diagnosis. *A previous prescription for zoster is defined as zoster/PHN code and prescription (TCA or strong painkiller) on the same day, 0-89 days after zoster

YES (n=762)

YES (n=772)

First ever non-specific neuralgia code 90-365 days after HZ

**NEW** anticonvulsant/ capsaicin cream/lidocaine patch, 90-180days after zoster

**NEW** TCA, 90-180 days after zoster with no other indication present AND a previous* TCA prescription for zoster

**NEW** strong painkiller, 90-180 days after zoster with no other indication present AND a previous* strong painkiller prescription for zoster

**NEW** TCA, 90-180 days after zoster with no other indication present

Neuropathic pain / peripheral neuropathy, 90-365 days after zoster

Zoster code and a zoster medication on same day, 90-365 days after zoster

PHN code 90-365 days after zoster in CPRD or HES

Exclusions N=25,556

*History of epilepsy prior to zoster diagnosis: n=2395*

*History of neuropathy prior to zoster diagnosis: n=1857*

*Less than 365 days follow-up following zoster: n=21,294*

YES (n=1237)

YES (n=918)

Possible PHN:

N=1,793

YES (n=113)

YES (n=783)

YES (n=215)

Final zoster cohort for analysis: N=119,413

Diagnosed PHN:

N=2,156

YES (n=2156)

**Figure e-2:** Prevalence of postherpetic neuralgia in the cohort of 119,413 zoster patients, by age group and postherpetic neuralgia classification (diagnosed, probable or possible postherpetic neuralgia)

N=1596

N=12063

N=22978

N=26065

N=23701

N=24282

N=8728

PHN: Postherpetic neuralgia. See Box 1 for description of PHN classifications.

**Figure e-3:** Association between postherpetic neuralgia and age at zoster diagnosis, modelled using a 5-knot restricted cubic spline

The 5-knot restricted cubic spline model is centred at 18 years. Knots were placed at equal percentiles of the data, with Akaike information criterion used to select optimal knots. Curves estimated were adjusted for gender, socioeconomic status, HIV, leukaemia, lymphoma, myeloma, hematopoietic stem cell transplantation other unspecified cellular immune deficiencies, immunosuppressive therapies, rheumatoid arthritis, systemic lupus erythematosis, inflammatory bowel disease, COPD, asthma, chronic kidney disease, depression, personality disorder, diabetes, recent cancer diagnosis, smoking, BMI category, antiviral use and site of zoster (as in Model 2 from table 2). The data are shown on a natural-log scale. See Appendix, section A3, for more detailed explanation of modelling age using splines.

 **Figure e-4:** Estimated associations between risk factors and PHN, with minimum number of days follow-up required lowered to reduce excluded patients

ORs are adjusted for age (modelled as a 5-knot restricted cubic spline to allow for non-linearity unless otherwise specified¹), gender, socioeconomic status, HIV, leukaemia, lymphoma, myeloma, hematopoietic stem cell transplantation, other unspecified cellular immune deficiencies, immunosuppressive therapies, rheumatoid arthritis, systemic lupus erythematosis, inflammatory bowel disease, COPD, asthma, chronic kidney disease, depression, personality disorder, diabetes, recent cancer diagnosis, smoking, BMI category, site of zoster and antiviral use. Note: The main analysis includes only patients followed up for at least 365 days following rash onset (Risk of PHN 5.8% (6956/119413). ¹Age not modelled as a 5-knot restricted cubic spline but included as a linear effect, with slopes changing at age 50 and 81. The two sensitivity analyses lowered the restrictions on follow-up time, so all patients followed up for 180 and 120 days were included in the analysis (Risk of PHN 4.9% (6345/129592) and 3.1% (4145/133312) respectively). Please note the y axis are not identical for all graphs. OID=other immune deficiencies. COPD=Chronic obstructive pulmonary disorder. HSCT=hematopoietic stem cell transplantation.

| Table e-1: Relative risk of PHN in patients with risk factors of interest stratified by age of diagnosis | | | | | | | | | |
| --- | --- | --- | --- | --- | --- | --- | --- | --- | --- |
|  | **<70 years** | | | | **≥70 years** | | | |  |
|  | **Total cohort, n** | **Risk of PHN, n (%)** | **Age-adjusted OR (99% CI)** | **Fully adjusted† OR (99% CI)** | **Total cohort, n** | **Risk of PHN, n (%)** | **Age-adjusted OR (99% CI)** | **Fully adjusted† OR (99% CI)** | **P-value for interaction** |
| Total | 82776 (100) | 2959 (3.6) |  |  | 36637 (100) | 3997 (10.9) |  |  |  |
| *Demographic characteristics* |  |  |  |  |  |  |  |  |  |
| Female | 48215 (58.2) | 1850 (3.8) | 1.20 (1.08-1.32) | 1.19 (1.07-1.32) | 22948 (62.6) | 2639 (11.5) | 1.14 (1.04-1.25) | 1.18 (1.07-1.31) | 0.872 |
| Socioeconomic status (practice level)¹ | |  |  |  |  |  |  |  |  |
| 1 (least deprived) | 16525 (20.0) | 528 (3.2) | 1.00 | 1.00 | 7533 (20.6) | 754 (10.0) | 1.00 | 1.00 | 0.811 |
| 2 | 16625 (20.1) | 563 (3.4) | 1.07 (0.89-1.29) | 1.08 (0.89-1.30) | 7028 (19.2) | 725 (10.3) | 1.04 (0.89-1.20) | 1.02 (0.87-1.19) |  |
| 3 | 16718 (20.2) | 581 (3.5) | 1.08 (0.90-1.30) | 1.08 (0.89-1.29) | 7850 (21.4) | 862 (11.0) | 1.10 (0.96-1.27) | 1.11 (0.96-1.29) |  |
| 4 | 17210 (20.8) | 662 (3.8) | 1.22 (1.02-1.46) | 1.17 (0.98-1.40) | 7784 (21.2) | 895 (11.5) | 1.17 (1.02-1.35) | 1.16 (1.00-1.34) |  |
| 5 (most deprived) | 15698 (19.0) | 625 (4.0) | 1.31 (1.10-1.58) | 1.22 (1.01-1.46) | 6442 (17.6) | 761 (11.8) | 1.22 (1.05-1.41) | 1.18 (1.01-1.38) |  |
|  |  |  |  |  |  |  |  |  |  |
| *Severe Immunosuppression* |  |  |  |  |  |  |  |  |  |
| HIV | 95 (0.1) | 4 (4.2) | 1.78 (0.47-6.75) | 1.73 (0.43-6.93) | 4 (0.0) | 2 (50.0) | 9.52 (0.71-127.82) | 5.09 (0.21-121.53) | 0.667 |
| Leukaemia | 92 (0.1) | 4 (4.3) | 1.09 (0.29-4.13) | 1.11 (0.29-4.27) | 61 (0.2) | 17 (27.9) | 3.26 (1.55-6.86) | 2.84 (1.31-6.16) | 0.234 |
| Lymphoma | 222 (0.3) | 25 (11.3) | 3.10 (1.77-5.42) | 3.08 (1.72-5.51) | 92 (0.3) | 15 (16.3) | 1.67 (0.80-3.47) | 1.88 (0.87-4.06) | 0.407 |
| Myeloma | 149 (0.2) | 21 (14.1) | 3.17 (1.71-5.89) | 2.95 (1.55-5.64) | 163 (0.4) | 32 (19.6) | 1.97 (1.18-3.29) | 1.96 (1.15-3.32) | 0.385 |
| Other unspecified cellular immune deficiencies | 37 (0.0) | 3 (8.1) | 2.18 (0.45-10.62) | 1.70 (0.34-8.46) | 23 (0.1) | 5 (21.7) | 2.23 (0.60-8.28) | 2.49 (0.65-9.48) | 0.835 |
| Oral corticosteroids² | 233 (0.3) | 24 (10.3) | 2.76 (1.56-4.86) | 2.14 (1.19-3.87) | 132 (0.4) | 29 (22.0) | 2.32 (1.34-4.01) | 2.26 (1.29-3.96) | 0.866 |
| Other immunosuppressive therapy² | 1174 (1.4) | 77 (6.6) | 1.69 (1.24-2.31) | 1.28 (0.89-1.84) | 534 (1.5) | 68 (12.7) | 1.26 (0.89-1.76) | 1.09 (0.73-1.62) | 0.261 |
|  |  |  |  |  |  |  |  |  |  |
| *Autoimmune conditions* |  |  |  |  |  |  |  |  |  |
| Rheumatoid Arthritis | 1380 (1.7) | 98 (7.1) | 1.61 (1.22-2.13) | 1.29 (0.94-1.76) | 1094 (3.0) | 127 (11.6) | 1.09 (0.85-1.40) | 1.02 (0.77-1.36) | 0.110 |
| Systemic Lupus Erythematosis | 226 (0.3) | 16 (7.1) | 2.23 (1.13-4.40) | 1.74 (0.87-3.50) | 81 (0.2) | 13 (16.0) | 1.65 (0.75-3.62) | 1.68 (0.76-3.73) | 0.826 |
| Inflammatory Bowel Disease | 1051 (1.3) | 54 (5.1) | 1.41 (0.97-2.03) | 1.19 (0.80-1.76) | 418 (1.1) | 56 (13.4) | 1.28 (0.88-1.86) | 1.26 (0.86-1.85) | 0.863 |
|  |  |  |  |  |  |  |  |  |  |
| *Other comorbidities* |  |  |  |  |  |  |  |  |  |
| COPD | 1995 (2.4) | 208 (10.4) | 2.10 (1.72-2.57) | 1.83 (1.49-2.26) | 3065 (8.4) | 461 (15.0) | 1.51 (1.31-1.73) | 1.41 (1.22-1.64) | 0.006 |
| Asthma | 6206 (7.5) | 276 (4.4) | 1.37 (1.16-1.63) | 1.33 (1.12-1.58) | 2061 (5.6) | 236 (11.5) | 1.08 (0.89-1.29) | 1.08 (0.89-1.31) | 0.129 |
| Chronic Kidney Disease | 1891 (2.3) | 124 (6.6) | 1.39 (1.08-1.78) | 1.18 (0.92-1.53) | 4098 (11.2) | 511 (12.5) | 1.13 (0.99-1.29) | 1.06 (0.93-1.22) | 0.290 |
| Depression | 4283 (5.2) | 204 (4.8) | 1.61 (1.32-1.96) | 1.42 (1.16-1.74) | 1176 (3.2) | 176 (15.0) | 1.44 (1.16-1.79) | 1.34 (1.07-1.69) | 0.548 |
| Personality disorder | 609 (0.7) | 33 (5.4) | 1.56 (0.98-2.50) | 1.34 (0.83-2.18) | 165 (0.5) | 20 (12.1) | 1.12 (0.60-2.07) | 1.07 (0.56-2.07) | 0.572 |
| Diabetes | 4357 (5.3) | 298 (6.8) | 1.53 (1.30-1.81) | 1.38 (1.16-1.65) | 4135 (11.3) | 491 (11.9) | 1.13 (0.99-1.29) | 1.09 (0.95-1.25) | 0.004 |
| Recent cancer diagnosis | 625 (0.8) | 43 (6.9) | 1.50 (0.99-2.27) | 1.40 (0.90-2.17) | 578 (1.6) | 54 (9.3) | 0.83 (0.57-1.21) | 0.88 (0.60-1.28) | 0.106 |
|  |  |  |  |  |  |  |  |  |  |
| *Health behaviours and characteristics of zoster episode* | | |  |  |  |  |  |  |  |
| Smoking |  |  |  |  |  |  |  |  |  |
| Non-smoker | 31837 (38.5) | 899 (2.8) | 1.00 | 1.00 | 13938 (38.0) | 1427 (10.2) | 1.00 | 1.00 | 0.006 |
| Current smoker | 24698 (29.8) | 962 (3.9) | 1.48 (1.31-1.68) | 1.39 (1.22-1.59) | 6058 (16.5) | 684 (11.3) | 1.17 (1.03-1.33) | 1.12 (0.97-1.28) |  |
| Ex-smoker | 25414 (30.7) | 1083 (4.3) | 1.28 (1.14-1.45) | 1.18 (1.04-1.34) | 16317 (44.5) | 1863 (11.4) | 1.15 (1.05-1.27) | 1.10 (0.99-1.23) |  |
| BMI Category |  |  |  |  |  |  |  |  |  |
| Underweight (BMI <18.5) | 1237 ( 1.5) | 60 ( 4.9) | 1.66 (1.16-2.37) | 1.42 (0.99-2.05) | 913 ( 2.5) | 128 (14.0) | 1.27 (0.98-1.64) | 1.18 (0.91-1.53) | 0.037 |
| Normal Weight (BMI 18.5-24.9) | 28856 (34.9) | 960 ( 3.3) | 1.00 | 1.00 | 13195 (36.0) | 1438 (10.9) | 1.00 | 1.00 |  |
| Overweight (BMI 25-30) | 26905 (32.5) | 979 ( 3.6) | 0.95 (0.84-1.07) | 0.97 (0.86-1.09) | 13029 (35.6) | 1416 (10.9) | 1.02 (0.92-1.13) | 1.04 (0.94-1.16) |  |
| Obese (BMI ≥30) | 17813 (21.5) | 829 ( 4.7) | 1.24 (1.10-1.41) | 1.17 (1.02-1.33) | 6436 (17.6) | 731 (11.4) | 1.11 (0.98-1.26) | 1.08 (0.95-1.22) |  |
| Antiviral record within 7 days of zoster | 46332 (56.0) | 1775 (3.8) | 1.08 (0.97-1.19) | 1.05 (0.95-1.16) | 22550 (61.6) | 2473 (11.0) | 1.03 (0.94-1.12) | 1.02 (0.93-1.12) | 0.800 |
| Anatomical site of zoster |  |  |  |  |  |  |  |  |  |
| Site Unspecified | 78915 (95.3) | 2684 (3.4) | 1.00 | 1.00 | 34085 (93.0) | 3530 (10.4) | 1.00 | 1.00 | 0.901 |
| Non-Truncal (excluding ophthalmic zoster) | 40 (1.4) | 40 (7.1) | 2.54 (1.64-3.92) | 2.31 (1.45-3.68) | 34 (0.9) | 34 (19.5) | 2.13 (1.29-3.51) | 2.04 (1.20-3.47) |  |
| Ophthalmic zoster | 235 (7.9) | 235 (7.1) | 1.94 (1.61-2.33) | 1.91 (1.58-2.31) | 433 (10.8) | 433 (18.2) | 1.91 (1.65-2.21) | 1.96 (1.68-2.28) |  |
| †Adjusted for age (modelled as a 5-knot restricted cubic spline to allow for non-linearity unless otherwise specified), gender, socioeconomic status, HIV, leukaemia, lymphoma, myeloma, other unspecified cellular immune deficiencies, immunosuppressive therapies, rheumatoid arthritis, systemic lupus erythematosis, inflammatory bowel disease, COPD, asthma, chronic kidney disease, depression, personality disorder, diabetes, recent cancer diagnosis, smoking, BMI category, site of zoster and antiviral use. Interaction terms between age and other risk factors were added one at a time into the multivariable regression model. ¹Measured by Index of Multiple deprivation score. ²Includes patients currently taking a 14 day course of immunosuppressive medications, or terminating a 14 day course of immunosuppressive medications less than one month prior to the zoster diagnosis. Oral corticosteroid prescriptions were required to be high dose (≥20mg per day). | | | | | | | | |  |

| Table e-2: Relative risk of PHN in patients with risk factors of interest stratified by age of diagnosis | | | | | | | | | |
| --- | --- | --- | --- | --- | --- | --- | --- | --- | --- |
|  | **<60 years** | | | | **≥60 years** | | | |  |
|  | **Total cohort, n** | **Risk of PHN, n (%)** | **Age-adjusted OR (99% CI)** | **Fully adjusted† OR (99% CI)** | **Total cohort, n** | **Risk of PHN, n (%)** | **Age-adjusted OR (99% CI)** | **Fully adjusted† OR (99% CI)** | **P-value for interaction** |
| Total | 56711 (100) | 1377 (2.4) |  |  | 62702 (100) | 5579 (8.9) |  |  |  |
| *Demographic characteristics* |  |  |  |  |  |  |  |  |  |
| Female | 33271 (58.7) | 917 (2.8) | 1.35 (1.16-1.56) | 1.31 (1.12-1.54) | 37892 (60.4) | 3572 (9.4) | 1.12 (1.04-1.21) | 1.16 (1.07-1.26) | 0.141 |
| Socioeconomic status (practice level)¹ | |  |  |  |  |  |  |  |  |
| 1 (least deprived) | 11204 (19.8) | 236 (2.1) | 1.00 | 1.00 | 12854 (20.5) | 1046 (8.1) | 1.00 | 1.00 | 0.898 |
| 2 | 11425 (20.1) | 265 (2.3) | 1.11 (0.86-1.44) | 1.11 (0.86-1.45) | 12228 (19.5) | 1023 (8.4) | 1.04 (0.91-1.19) | 1.03 (0.89-1.18) |  |
| 3 | 11281 (19.9) | 265 (2.3) | 1.13 (0.87-1.46) | 1.12 (0.86-1.45) | 13287 (21.2) | 1178 (8.9) | 1.09 (0.95-1.24) | 1.09 (0.95-1.25) |  |
| 4 | 11757 (20.7) | 308 (2.6) | 1.26 (0.98-1.62) | 1.19 (0.93-1.54) | 13237 (21.1) | 1249 (9.4) | 1.18 (1.03-1.34) | 1.16 (1.01-1.33) |  |
| 5 (most deprived) | 11044 (19.5) | 303 (2.7) | 1.37 (1.06-1.76) | 1.24 (0.96-1.61) | 11096 (17.7) | 1083 (9.8) | 1.24 (1.08-1.42) | 1.19 (1.04-1.37) |  |
|  |  |  |  |  |  |  |  |  |  |
| *Severe Immunosuppression* |  |  |  |  |  |  |  |  |  |
| HIV | 90 (0.2) | 4 (4.4) | 2.08 (0.55-7.88) | 2.38 (0.61-9.31) | 9 (0.0) | 2 (22.2) | 3.55 (0.44-28.86) | 1.60 (0.10-26.75) | 0.635 |
| Leukaemia | 55 (0.1) | 2 (3.6) | 1.34 (0.21-8.74) | 1.29 (0.19-8.61) | 98 (0.2) | 19 (19.4) | 2.53 (1.30-4.94) | 2.21 (1.10-4.44) | 0.554 |
| Lymphoma | 128 (0.2) | 13 (10.2) | 4.46 (2.07-9.63) | 4.32 (1.95-9.54) | 186 (0.3) | 27 (14.5) | 1.91 (1.11-3.29) | 2.04 (1.15-3.62) | 0.117 |
| Myeloma | 56 (0.1) | 6 (10.7) | 3.61 (1.17-11.18) | 3.63 (1.12-11.74) | 256 (0.4) | 47 (18.4) | 2.23 (1.46-3.41) | 2.16 (1.40-3.34) | 0.358 |
| Other unspecified cellular immune deficiencies | 20 (0.0) | 2 (10.0) | 5.21 (0.74-36.80) | 4.21 (0.55-31.95) | 40 (0.1) | 6 (15.0) | 1.80 (0.57-5.72) | 1.78 (0.55-5.71) | 0.410 |
| Oral corticosteroids² | 140 (0.2) | 12 (8.6) | 3.46 (1.57-7.63) | 2.66 (1.17-6.02) | 225 (0.4) | 41 (18.2) | 2.33 (1.48-3.66) | 2.10 (1.32-3.36) | 0.474 |
| Other immunosuppressive therapy² | 691 (1.2) | 31 (4.5) | 1.71 (1.06-2.78) | 1.15 (0.65-2.02) | 1017 (1.6) | 114 (11.2) | 1.39 (1.07-1.81) | 1.18 (0.87-1.60) | 0.479 |
|  |  |  |  |  |  |  |  |  |  |
| *Autoimmune conditions* |  |  |  |  |  |  |  |  |  |
| Rheumatoid Arthritis | 659 (1.2) | 33 (5.0) | 1.73 (1.08-2.78) | 1.37 (0.81-2.31) | 1815 (2.9) | 192 (10.6) | 1.21 (0.99-1.48) | 1.09 (0.87-1.38) | 0.263 |
| Systemic Lupus Erythematosis | 170 (0.3) | 8 (4.7) | 1.92 (0.75-4.94) | 1.41 (0.54-3.73) | 137 (0.2) | 21 (15.3) | 1.95 (1.05-3.63) | 1.86 (0.99-3.49) | 0.652 |
| Inflammatory Bowel Disease | 713 (1.3) | 28 (3.9) | 1.56 (0.94-2.58) | 1.35 (0.79-2.32) | 756 (1.2) | 82 (10.8) | 1.27 (0.94-1.73) | 1.18 (0.86-1.63) | 0.559 |
|  |  |  |  |  |  |  |  |  |  |
| *Other comorbidities* |  |  |  |  |  |  |  |  |  |
| COPD | 549 (1.0) | 32 (5.8) | 1.75 (1.08-2.84) | 1.44 (0.88-2.35) | 4511 (7.2) | 637 (14.1) | 1.66 (1.48-1.87) | 1.54 (1.36-1.74) | 0.675 |
| Asthma | 4449 (7.8) | 150 (3.4) | 1.52 (1.21-1.92) | 1.40 (1.10-1.77) | 3818 (6.1) | 362 (9.5) | 1.12 (0.97-1.30) | 1.14 (0.97-1.32) | 0.068 |
| Chronic Kidney Disease | 773 (1.4) | 41 (5.3) | 1.92 (1.25-2.93) | 1.59 (1.02-2.48) | 5216 (8.3) | 594 (11.4) | 1.14 (1.01-1.29) | 1.06 (0.93-1.20) | 0.018 |
| Depression | 3407 (6.0) | 127 (3.7) | 1.67 (1.31-2.14) | 1.43 (1.11-1.85) | 2052 (3.3) | 253 (12.3) | 1.47 (1.23-1.75) | 1.36 (1.13-1.64) | 0.427 |
| Personality disorder | 424 (0.7) | 21 (5.0) | 2.04 (1.13-3.67) | 1.66 (0.91-3.05) | 350 (0.6) | 32 (9.1) | 1.11 (0.68-1.79) | 1.05 (0.64-1.74) | 0.180 |
| Diabetes | 1949 (3.4) | 98 (5.0) | 1.78 (1.34-2.35) | 1.47 (1.09-1.97) | 6543 (10.4) | 691 (10.6) | 1.20 (1.08-1.35) | 1.16 (1.03-1.30) | 0.018 |
| Recent cancer diagnosis | 283 (0.5) | 14 (4.9) | 1.68 (0.82-3.43) | 1.41 (0.65-3.06) | 920 (1.5) | 83 (9.0) | 0.98 (0.72-1.32) | 1.01 (0.74-1.38) | 0.380 |
|  |  |  |  |  |  |  |  |  |  |
| *Health behaviours and characteristics of zoster episode* | | |  |  |  |  |  |  |  |
| Smoking |  |  |  |  |  |  |  |  |  |
| Non-smoker | 22730 (40.1) | 457 (2.0) | 1.00 | 1.00 | 23045 (36.8) | 1869 (8.1) | 1.00 | 1.00 | 0.324 |
| Current smoker | 18413 (32.5) | 506 (2.7) | 1.43 (1.21-1.70) | 1.39 (1.16-1.67) | 12343 (19.7) | 1140 (9.2) | 1.29 (1.16-1.43) | 1.21 (1.09-1.36) |  |
| Ex-smoker | 14829 (26.1) | 405 (2.7) | 1.22 (1.02-1.46) | 1.15 (0.95-1.38) | 26902 (42.9) | 2541 (9.4) | 1.20 (1.10-1.31) | 1.13 (1.04-1.24) |  |
| BMI Category |  |  |  |  |  |  |  |  |  |
| Underweight (BMI <18.5) | 909 (1.6) | 29 (3.2) | 1.76 (1.06-2.93) | 1.58 (0.95-2.64) | 1241 (2.0) | 159 (12.8) | 1.31 (1.04-1.65) | 1.19 (0.94-1.50) | 0.031 |
| Normal Weight (BMI 18.5-24.9) | 20643 (36.4) | 457 (2.2) | 1.00 | 1.00 | 21408 (34.1) | 1941 (9.1) | 1.00 | 1.00 |  |
| Overweight (BMI 25-30) | 16932 (29.9) | 419 (2.5) | 1.00 (0.84-1.19) | 1.03 (0.86-1.23) | 23002 (36.7) | 1976 (8.6) | 0.99 (0.90-1.08) | 1.01 (0.92-1.10) |  |
| Obese (BMI ≥30) | 11506 (20.3) | 396 (3.4) | 1.39 (1.16-1.66) | 1.29 (1.07-1.56) | 12743 (20.3) | 1164 (9.1) | 1.12 (1.01-1.24) | 1.08 (0.97-1.20) |  |
| Antiviral record within 7 days of zoster | 30510 (53.8) | 796 (2.6) | 1.10 (0.96-1.28) | 1.06 (0.91-1.23) | 38372 (61.2) | 3452 (9.0) | 1.03 (0.96-1.11) | 1.03 (0.95-1.11) | 0.548 |
| Anatomical site of zoster |  |  |  |  |  |  |  |  |  |
| Site Unspecified | 54309 (95.8) | 1262 (2.3) | 1.00 | 1.00 | 58691 (93.6) | 4952 (8.4) | 1.00 | 1.00 | 0.789 |
| Non-Truncal (excluding ophthalmic zoster) | 425 (0.7) | 21 (4.9) | 2.38 (1.32-4.29) | 1.94 (1.01-3.73) | 311 (0.5) | 53 (17.0) | 2.31 (1.56-3.44) | 2.27 (1.49-3.46) |  |
| Ophthalmic zoster | 1977 (3.5) | 94 (4.8) | 1.94 (1.46-2.57) | 1.92 (1.43-2.59) | 3700 (5.9) | 574 (15.5) | 1.92 (1.69-2.17) | 1.94 (1.71-2.21) |  |
| †Adjusted for age (modelled as a 5-knot restricted cubic spline to allow for non-linearity unless otherwise specified), gender, socioeconomic status, HIV, leukaemia, lymphoma, myeloma, other unspecified cellular immune deficiencies, immunosuppressive therapies, rheumatoid arthritis, systemic lupus erythematosis, inflammatory bowel disease, COPD, asthma, chronic kidney disease, depression, personality disorder, diabetes, recent cancer diagnosis, smoking, BMI category, site of zoster and antiviral use. Interaction terms between age and other risk factors were added one at a time into the multivariable regression model. ¹Measured by Index of Multiple deprivation score. ²Includes patients currently taking a 14 day course of immunosuppressive medications, or terminating a 14 day course of immunosuppressive medications less than one month prior to the zoster diagnosis. Oral corticosteroid prescriptions were required to be high dose (≥20mg per day). | | | | | | | | |  |

| Table e-3: Sensitivity analysis using alternative definitions of PHN | | | | | | | |
| --- | --- | --- | --- | --- | --- | --- | --- |
|  | **PRIMARY PHN definition (results from Model 2)** | **PHN defined as pain at 30 days after zoster*** | | **PHN restricted to diagnosed cases only** | | **Excluding patients possible herpes simplex patients (n=1586)** | |
|  | **Fully adjusted OR† (99% CI)** | **Risk of PHN, n (%)** | **Fully adjusted OR† (99% CI)** | **Risk of PHN, n (%)** | **Fully adjusted OR† (99% CI)** | **Risk of PHN, n (%)** | **Fully adjusted**  **OR† (99% CI)** |
| Total | - | 11755/119413 (9.8) | - | 2156/119413 (1.8) |  | 6810/117827 (5.8) |  |
| *Demographic characteristics* |  |  |  |  |  |  |  |
| Age (years) [Estimated ORs for 10 year increase in age within the specified age-band]¹ | | | | |  |  |  |
| <50 | 1.42 (1.28-1.57) | 922 (2.8) | 1.54 (1.42-1.67) | 127 (0.4) | 1.36 (1.10-1.68) | 569 (1.8) | 1.41 (1.27-1.56) |
| 50-79 | 1.70 (1.63-1.78) | 8104 (11.1) | 1.73 (1.67-1.78) | 1396 (1.9) | 2.08 (1.92-2.24) | 4530 (6.3) | 1.71 (1.64-1.79) |
| ≥80 | 1.10 (0.94-1.28) | 2729 (20.0) | 0.96 (0.85-1.09) | 633 (4.6) | 0.89 (0.69-1.15) | 1711 (12.7) | 1.10 (0.95-1.28) |
| Female | 1.19 (1.10-1.27) | 7467 (10.5) | 1.15 (1.09-1.22) | 1332 (1.9) | 1.00 (0.89-1.14) | 4382 (6.3)) | 1.18 (1.10-1.27) |
| Socioeconomic status (practice level)² |  |  |  |  |  |  |  |
| 1 (least deprived) | 1.00 | 2208 (9.2) | 1.00 | 461 (1.9) | 1.00 | 1259 (5.3) | 1.00 |
| 2 | 1.04 (0.91-1.18) | 2217 (9.4) | 1.04 (0.93-1.15) | 401 (1.7) | 0.94 (0.70-1.25) | 1257 (5.4) | 1.03 (0.91-1.18) |
| 3 | 1.09 (0.96-1.24) | 2461 (10.0) | 1.09 (0.98-1.21) | 430 (1.8) | 0.89 (0.67-1.19) | 1415 (5.8) | 1.09 (0.96-1.24) |
| 4 | 1.17 (1.03-1.32) | 2560 (10.2) | 1.13 (1.02-1.25) | 477 (1.9) | 0.99 (0.75-1.32) | 1529 (6.2) | 1.17 (1.03-1.33) |
| 5 (most deprived) | 1.20 (1.06-1.37) | 2309 (10.4) | 1.18 (1.06-1.31) | 387 (1.7) | 0.98 (0.74-1.32) | 1350 (6.2) | 1.19 (1.04-1.36) |
| *Severe Immunosuppression* |  |  |  |  |  |  |  |
| HIV | 2.17 (0.64-7.37) | 11 (11.1) | 2.86 (1.16-7.07) | 2 (2.0) | 1.71(0.12-24.01) | 6 (6.2) | 2.22 (0.65-7.56) |
| Leukaemia | 2.07 (1.08-3.96) | 34 (22.2) | 2.11 (1.22-3.64) | 7 (4.6) | 2.07 (0.73-5.87) | 21 (14.4) | 2.27 (1.18-4.36) |
| Lymphoma | 2.45 (1.53-3.92) | 61 (19.4) | 2.27 (1.52-3.40) | 10 (3.2) | 1.94 (0.79-4.75) | 37 (12.1) | 2.34 (1.43-3.82) |
| Myeloma | 2.17 (1.43-3.29) | 79 (25.3) | 2.06 (1.44-2.95) | 19 (6.1) | 2.33 (1.21-4.46) | 52 (17.2) | 2.20 (1.44-3.36) |
| Hematopoietic stem cell transplantation | 5.91 (1.32-26.59) | 6 (35.3) | 4.72 (1.12-19.77) | 0 (0.0) | - | 5 (31.3) | 6.89 (1.48-32.02) |
| Other unspecified cellular immune deficiencies | 2.12 (0.77-5.89) | 11 (18.3) | 1.79 (0.72-4.43) | 0 (0.0) | - | 8 (13.6) | 2.14 (0.77-5.92) |
| Oral corticosteroids³ | 2.26 (1.51-3.40) | 79 (21.6) | 2.08 (1.46-2.96)) | 20 (5.5) | 2.83 (1.50-5.33) | 53 (14.6) | 2.31 (1.54-3.47) |
| Other immunosuppressive therapy³ | 1.21 (0.92-1.58) | 239 (14.0) | 1.24 (1.00-1.54) | 40 (2.3) | 1.14 (0.70-1.86) | 144 (8.6) | 1.23 (0.94-1.61) |
| *Autoimmune conditions* |  |  |  |  |  |  |  |
| Rheumatoid Arthritis | 1.13 (0.91-1.39) | 363 (14.7) | 1.09 (0.92-1.29) | 66 (2.7) | 1.04 (0.71-1.52) | 223 (9.1) | 1.14 (0.92-1.41) |
| Systemic Lupus Erythematosis | 1.76 (1.04-2.98) | 40 (13.0) | 1.32 (0.82-2.12) | 12 (3.9) | 2.66 (1.20-5.88) | 29 (9.6) | 1.80 (1.07-3.05) |
| Inflammatory Bowel Disease | 1.22 (0.93-1.60) | 173 (11.8) | 1.13 (0.90-1.42) | 26 (1.8) | 0.91 (0.53-1.56) | 108 (7.5) | 1.22 (0.92-1.61) |

| Table e-3: Sensitivity analysis using alternative definitions of PHN (continued) | | | | | | | |
| --- | --- | --- | --- | --- | --- | --- | --- |
|  | **Final analysis for PRIMARY definition** | **PHN defined as pain at 30 days after zoster*** | | **PHN restricted to diagnosed** | | **Excluding patients with zoster code following first zoster diagnosis (possible herpes simplex)** | |
|  | **Fully adjusted OR (99% CI)²** | **Risk of PHN 30, n (%)** | **Fully adjusted OR² (99% CI)** | **Risk of PHN, n (%)** | **Risk of PHN, n (%)** | **Risk of PHN, n (%)** | **Fully adjusted OR² (99% CI)** |
| *Other comorbidities* |  |  |  |  |  |  |  |
| COPD | 1.53 (1.35-1.72) | 1054 (20.8) | 1.49 (1.35-1.65) | 219 (4.3) | 1.43 (1.17-1.76) | 654 (13.1) | 1.52 (1.34-1.72) |
| Asthma | 1.21 (1.06-1.37) | 866 (10.5) | 1.24 (1.12-1.37) | 147 (1.8) | 1.17 (0.93-1.48) | 503 (6.2) | 1.22 (1.07-1.38) |
| Chronic Kidney Disease | 1.08 (0.96-1.22) | 1006 (16.8) | 1.05 (0.95-1.16) | 176 (2.9) | 0.91 (0.73-1.13) | 621 (10.5) | 1.07 (0.95-1.21) |
| Depression | 1.40 (1.20-1.62) | 575 (10.5) | 1.27 (1.12-1.45) | 93 (1.7) | 1.12 (0.84-1.49) | 372 (7.0) | 1.41 (1.21-1.64) |
| Personality disorder | 1.25 (0.85-1.85) | 76 (9.8) | 1.04 (0.74-1.46) | 17 (2.2) | 1.48 (0.77-2.84) | 52 (6.8) | 1.28 (0.87-1.90) |
| Diabetes | 1.19 (1.07-1.33) | 1300 (15.3) | 1.19 (1.09-1.29) | 205 (2.4) | 0.94 (0.77-1.15) | 773 (9.2) | 1.18 (1.06-1.32) |
| Recent cancer diagnosis | 1.06 (0.79-1.41) | 156 (13.0) | 0.98 (0.78-1.25) | 26 (2.2) | 0.89 (0.53-1.51) | 93 (7.9) | 1.03 (0.77-1.39) |
|  |  |  |  |  |  |  |  |
| *Health behaviours and characteristics of zoster episode* | |  |  |  |  |  |  |
| Smoking |  |  |  |  |  |  |  |
| Non-smoker | 1.00 | 3978 (8.7) | 1.00 | 743 (1.6) | 1.00 | 2274 (5.0) | 1.00 |
| Current smoker | 1.27 (1.15-1.39) | 2800 (9.1) | 1.26 (1.17-1.36) | 491 (1.6) | 1.19 (1.00-1.40) | 1610 (5.3) | 1.27 (1.15-1.39) |
| Ex-smoker | 1.14 (1.05-1.24) | 4907 (11.8) | 1.11 (1.04-1.19) | 915 (2.2) | 1.09 (0.94-1.25) | 2891 (7.0) | 1.15 (1.06-1.24) |
| BMI Category |  |  |  |  |  |  |  |
| Underweight (BMI <18.5) | 1.25 (1.01-1.54) | 282 (13.1) | 1.15 (0.96-1.38) | 77 (3.6) | 1.56 (1.13-2.17) | 187 (8.8) | 1.27 (1.03-1.57) |
| Normal Weight (BMI 18.5-24.9) | 1.00 | 4026 (9.6) | 1.00 | 771 (1.8) | 1.00 | 2345 (5.7) | 1.00 |
| Overweight (BMI 25-30) | 1.01 (0.93-1.09) | 4131 (10.3) | 1.03 (0.97-1.10) | 749 (1.9) | 0.99 (0.86-1.14) | 2338 (5.9) | 1.01 (0.93-1.09) |
| Obese (BMI ≥30) | 1.13 (1.03-1.24) | 2570 (10.6) | 1.11 (1.03-1.19) | 425 (1.8) | 1.03 (0.87-1.21) | 1536 (6.4) | 1.14 (1.04-1.25) |
| Antiviral record within 7 days of zoster | 1.04 (0.97-1.11) | 7345 (10.7) | 1.12 (1.06-1.18) | 1355 (2.0) | 1.08 (0.96-1.22) | 4151 (6.1) | 1.04 (0.97-1.11) |
| Anatomical site of zoster |  |  |  |  |  |  |  |
| Site Unspecified | 1.00 | 10648 ( 9.4) | 1.00 | 1854 (1.6) | 1.00 | 6097 (5.5) | 1.00 |
| Non-Truncal (excluding ophthalmic zoster) | 2.19 (1.54-3.11) | 110 (15.0) | 2.00 (1.49-2.70) | 15 (2.0) | 1.77 (0.88-3.53) | 74 (10.5) | 2.34 (1.65-3.34) |
| Ophthalmic zoster | 1.95 (1.73-2.19) | 997 (17.6) | 1.72 (1.56-1.91) | 287 (5.1) | 2.67 (2.24-3.19) | 639 (11.6) | 1.94 (1.72-2.18) |
| *PHN at 30 days: same definition as PHN at 90 days, but gathering evidence from 30-365 days following zoster. †Adjusted for age (modelled as a 5-knot restricted cubic spline to allow for non-linearity unless otherwise specified), gender, socioeconomic status, HIV, leukaemia, lymphoma, myeloma, hematopoietic stem cell transplantation, other unspecified cellular immune deficiencies, immunosuppressive therapies, rheumatoid arthritis, systemic lupus erythematosis, inflammatory bowel disease, COPD, asthma, chronic kidney disease, depression, personality disorder, diabetes, recent cancer diagnosis, smoking, BMI category, site of zoster and antiviral use.¹ORs estimate the effect of a 10-year increase in age on PHN derived, in age groups <50, 50-79 and ≥80, from piecewise linear splines. ²Measured by Index of Multiple deprivation score. ³Includes patients currently taking a 14 day course of immunosuppressive medications, or terminating a 14 day course of immunosuppressive medications less than one month prior to the zoster diagnosis. Oral corticosteroid prescriptions were required to be high dose (≥20mg per day). | | | | | | | |

| Table e-4: Relative risk of diagnosed, probable or possible PHN by gender (N=119,413) | | | | | | |
| --- | --- | --- | --- | --- | --- | --- |
|  | **Diagnosed PHN** | | **Probable PHN** | | **Possible PHN** | |
|  | n (%) | Adjusted OR*  (99% CI) | n (%) | Adjusted OR*  (99% CI) | n (%) | Adjusted OR*  (99% CI) |
| Total | 2156 (1.8) |  | 3007 (2.5) |  | 1793 (1.5) |  |
| Male | 824 (1.7) | 1.00 | 1041 (2.2) | 1.00 | 602 (1.3) | 1.00 |
| Female | 1332 (1.9) | 1.01 (0.90-1.13) | 1966 (2.8) | 1.21 (1.09-1.33) | 1191 (1.7) | 1.27 (1.12-1.45) |

*Association between gender and PHN is age-adjusted (with age modelled as a 5 knot-restricted cubic spline).

| Table e-5: Association between female gender and PHN, according to different PHN outcome definitions | | | | | | | | |  |
| --- | --- | --- | --- | --- | --- | --- | --- | --- | --- |
|  | | | |  |  |  | | |  |
| PHN evidence category | | | | *ANALYSIS A:*  *Whole study cohort (N=119413)* | | | *ANALYSIS B:*  *Patients with a history of mental health problems (N=37451)* | *ANALYSIS C:*  *Patients with NO history of mental health problems*  *(N= 81962)* | *ANALYSIS D:*  *Patients with no evidence of depression one year before or after zoster*  *(N=* *110,730)* |
|  |  | | | Risk of specific PHN diagnosis (%) | Risk of specific PHN diagnosis in females n (%) | Age-adjusted OR (99% CI) | Age-adjusted OR (99% CI) | Age-adjusted OR (99% CI) | Age-adjusted OR (99% CI) |
| PHN |  | | |  |  |  |  |  |  |
| *Diagnosed* | PHN code* | | | 2156 (1.9) | 1332 (2.0) | 1.02 (0.91-1.15) | 1.02 (0.82-1.27) | 0.95 (0.82-1.10) | 1.01 (0.90-1.15) |
|  |  | | |  |  |  |  |  |  |
| *Presumed* | Zoster code and prescription same day* | | | 215 (0.2) | 146 (0.2) | 1.35 (0.92-1.97) | 1.41 (0.65-3.08) | 1.32 (0.85-2.06) | 1.33 (0.90-1.96) |
|  | Non-specific neuralgia code* | | | 783 (0.7) | 511 (0.7) | 1.21 (1.00-1.48) | 1.16 (0.80-1.67) | 1.19 (0.94-1.51) | 1.22 (1.00-1.50) |
|  | **NEW** anticonvulsant** | | | 1120 (0.9) | 699 (1.0) | 1.04 (0.89-1.23) | 0.87 (0.66-1.13) | 1.02 (0.83-1.26) | 1.04 (0.88-1.24) |
|  | **NEW** capsaicin cream/lidocaine patch** | | | 117 (0.1) | 75 (0.1) | 1.11 (0.67-1.84) | 1.58 (0.57-4.41) | 1.02 (0.83-1.26) | 1.11 (0.67-1.84) |
|  | **NEW** TCA ** with PHN/zoster code and zoster medication on the same day, 0-89 days after zoster | | | 772 (0.7) | 535 (0.8) | 1.43 (1.16-1.75) | 1.36 (0.95-1.96) | 1.34 (1.04-1.72) | 1.39 (1.13-1.72) |
|  |  | | |  |  |  |  |  |  |
| *Possible* | **NEW** TCA** | | | 918 (0.8) | 653 (0.9) | 1.55 (1.28-1.88) | 1.45 (1.04-2.01) | 1.42 (1.12-1.81) | 1.50 (1.23-1.84) |
|  | **NEW** strong PK ** with PHN/zoster code and zoster medication on the same day, 0-89 days after zoster | | | 762 (0.6) | 457 (0.6) | 0.98 (0.81-1.18) | 0.86 (0.60-1.24) | 1.00 (0.79-1.26) | 0.96 (0.79-1.17) |
|  | Non-specific neuropathy code* | | | 113 (0.09) | 81 (0.11) | 1.71 (1.00-2.93) | 1.55 (0.62-3.88) | 1.63 (0.83-3.21) | 1.84 (1.04-3.27) |
|  | |  | TCA: tricyclic antidepressant. PK: Painkiller. *90-365d post-zoster ** 90-180d post-zoster | | | | | |  |

| Table e-6: Estimated mean number of consultations per year during follow-up prior to zoster, for patients with each risk factor. | | |
| --- | --- | --- |
| Exposure | **Estimated mean number of consultations per year during follow-up, for patients with each exposure¹** | **Adjusted OR† (95% CI)PRIMARY PHN definition (results from Model 2)** |
| Overall | 7.3 | - |
| *Demographic characteristics* |  |  |
| Gender |  |  |
| Female | 8.1 | 1.19 (1.10-1.27) |
| Male | 6.0 | 1.00 |
| Socioeconomic status (practice level)² |  |  |
| 1 (least deprived) | 7.1 | 1.00 |
| 2 | 7.1 | 1.04 (0.91-1.18) |
| 3 | 7.2 | 1.09 (0.96-1.24) |
| 4 | 7.4 | 1.17 (1.03-1.32) |
| 5 (most deprived) | 7.5 | 1.20 (1.06-1.37) |
|  |  |  |
| *Severe Immunosuppression* |  |  |
| HIV | 7.5 | 2.17 (0.64-7.37) |
| Leukaemia | 8.8 | 2.07 (1.08-3.96) |
| Lymphoma | 8.5 | 2.45 (1.53-3.92) |
| Myeloma | 10.7 | 2.17 (1.43-3.29) |
| Hematopoietic stem cell transplantation | 9.6 | 5.91 (1.32-26.59) |
| Other unspecified cellular immune deficiencies | 12.5 | 2.12 (0.77-5.89) |
| Oral corticosteroids³ | 13.3 | 2.26 (1.51-3.40) |
| Other immunosuppressive therapy³ | 13.0 | 1.21 (0.92-1.58) |
|  |  |  |
| *Autoimmune conditions* |  |  |
| Rheumatoid Arthritis | 12.2 | 1.13 (0.91-1.39) |
| Systemic Lupus Erythematosis | 11.4 | 1.76 (1.04-2.98) |
| Inflammatory Bowel Disease | 10.6 | 1.22 (0.93-1.60) |
|  |  |  |
| *Other comorbidities* |  |  |
| COPD | 12.4 | 1.53 (1.35-1.72) |
| Asthma | 10.0 | 1.21 (1.06-1.37) |
| Chronic Kidney Disease | 11.4 | 1.08 (0.96-1.22) |
| Depression | 10.8 | 1.40 (1.20-1.62) |
| Personality disorder | 11.6 | 1.25 (0.85-1.85) |
| Diabetes | 12.2 | 1.19 (1.07-1.33) |
| Recent cancer diagnosis | 9.3 | 1.06 (0.79-1.41) |
|  |  |  |
| *Health behaviours and characteristics of zoster episode* | | |
| Smoking |  |  |
| Non-smoker | 6.6 | 1.00 |
| Current smoker | 6.8 | 1.27 (1.15-1.39) |
| Ex-smoker | 8.3 | 1.14 (1.05-1.24) |
| BMI Category |  |  |
| Underweight (BMI <18.5) | 8.0 | 1.25 (1.01-1.54) |
| Normal Weight (BMI 18.5-24.9) | 6.9 | 1.00 |
| Overweight (BMI 25-30) | 7.3 | 1.01 (0.93-1.09) |
| Obese (BMI ≥30) | 8.8 | 1.13 (1.03-1.24) |
| Antiviral record within 7 days of zoster | 7.4 | 1.04 (0.97-1.11) |
| Anatomical site of zoster |  |  |
| Site Unspecified | 7.2 | 1.00 |
| Non-Truncal (excluding ophthalmic zoster) | 8.9 | 2.19 (1.54-3.11) |
| Ophthalmic zoster | 7.9 | 1.95 (1.73-2.19) |
|  |  |  |
| CONTROL RISK FACTOR: Hypothyroidism | 10.9 | 1.01 (0.90-1.14) |
| †Adjusted for age (modelled as a 5-knot restricted cubic spline to allow for non-linearity), gender, socioeconomic status, HIV, leukaemia, lymphoma, myeloma, hematopoietic stem cell transplantation, other unspecified cellular immune deficiencies, immunosuppressive therapies, rheumatoid arthritis, systemic lupus erythematosis, inflammatory bowel disease, COPD, asthma, chronic kidney disease, depression, personality disorder, diabetes, recent cancer diagnosis, smoking, BMI category, site of zoster and antiviral use. ¹Calculated by dividing the total number of face-to-face or telephone consultations, by the total years of follow-up prior to zoster diagnosis for each patient and calculating the mean number of consultations per year for patients with each risk factor. ²Measured by Index of Multiple deprivation score. ³Includes patients currently taking a 14 day course of immunosuppressive medications, or terminating a 14 day course of immunosuppressive medications less than one month prior to the zoster diagnosis. Oral corticosteroid prescriptions were required to be high dose (≥20mg per day). | | |

| Table e-7: Relative risk of PHN in patients with various risk factors, using a multiply imputed dataset | | |
| --- | --- | --- |
| Exposure | **Adjusted OR† (95% CI) from multiply imputed dataset** | **Adjusted OR† (95% CI) PRIMARY PHN definition (results from Model 2)** |
| *Demographic characteristics* |  |  |
| Age (years) [Estimated ORs for 10 year increase in age within the specified age-band]¹ | | |
| <50 | 1.45 (1.32-1.60) | 1.42 (1.28-1.57)² |
| 50-79 | 1.71 (1.64-1.78) | 1.70 (1.63-1.78)² |
| ≥80 | 1.04 (0.90-1.19) | 1.10 (0.94-1.28)² |
| Female | 1.19 (1.11-1.28) | 1.19 (1.10-1.27) |
| Socioeconomic status (practice level)² |  |  |
| 1 (least deprived) |  | 1.00 |
| 2 | 1.04 (0.91-1.18) | 1.04 (0.91-1.18) |
| 3 | 1.07 (0.95-1.22) | 1.09 (0.96-1.24) |
| 4 | 1.16 (1.03-1.32) | 1.17 (1.03-1.32) |
| 5 (most deprived) | 1.20 (1.05-1.36) | 1.20 (1.06-1.37) |
|  |  |  |
| *Severe Immunosuppression* |  |  |
| HIV | 2.39 (0.78-7.33) | 2.17 (0.64-7.37) |
| Leukaemia | 2.11 (1.12-3.98) | 2.07 (1.08-3.96) |
| Lymphoma | 2.32 (1.47-3.66) | 2.45 (1.53-3.92) |
| Myeloma | 2.20 (1.46-3.30) | 2.17 (1.43-3.29) |
| Hematopoietic stem cell transplantation | 6.14 (1.36-27.66) | 5.91 (1.32-26.59) |
| Other unspecified cellular immune deficiencies | 1.93 (0.70-5.29) | 2.12 (0.77-5.89) |
| Oral corticosteroids³ | 2.25 (1.51-3.35) | 2.26 (1.51-3.40) |
| Other immunosuppressive therapy³ | 1.26 (0.97-1.63) | 1.21 (0.92-1.58) |
|  |  |  |
| *Autoimmune conditions* |  |  |
| Rheumatoid Arthritis | 1.10 (0.89-1.35) | 1.13 (0.91-1.39) |
| Systemic Lupus Erythematosis | 1.66 (0.99-2.80) | 1.76 (1.04-2.98) |
| Inflammatory Bowel Disease | 1.24 (0.95-1.62) | 1.22 (0.93-1.60) |
|  |  |  |
| *Other comorbidities* |  |  |
| COPD | 1.56 (1.39-1.76) | 1.53 (1.35-1.72) |
| Asthma | 1.23 (1.08-1.39) | 1.21 (1.06-1.37) |
| Chronic Kidney Disease | 1.10 (0.97-1.24) | 1.08 (0.96-1.22) |
| Depression | 1.41 (1.22-1.63) | 1.40 (1.20-1.62) |
| Personality disorder | 1.26 (0.86-1.83) | 1.25 (0.85-1.85) |
| Diabetes | 1.20 (1.08-1.34) | 1.19 (1.07-1.33) |
| Recent cancer diagnosis | 1.04 (0.78-1.37) | 1.06 (0.79-1.41) |
|  |  |  |
| *Health behaviours and characteristics of zoster episode* | | |
| Smoking |  |  |
| Non-smoker | 1.00 | 1.00 |
| Current smoker | 1.27 (1.16-1.39) | 1.27 (1.15-1.39) |
| Ex-smoker | 1.16 (1.07-1.25) | 1.14 (1.05-1.24) |
| BMI Category |  |  |
| Underweight (BMI <18.5) | 1.24 (0.99-1.56) | 1.25 (1.01-1.54) |
| Normal Weight (BMI 18.5-24.9) | 1.00 | 1.00 |
| Overweight (BMI 25-30) | 1.01 (0.94-1.10) | 1.01 (0.93-1.09) |
| Obese (BMI ≥30) | 1.14 (1.04-1.25) | 1.13 (1.03-1.24) |
| Antiviral record within 7 days of zoster | 1.04 (0.97-1.11) | 1.04 (0.97-1.11) |
| Anatomical site of zoster |  |  |
| Site Unspecified | 1.00 | 1.00 |
| Non-Truncal (excluding ophthalmic zoster) | 2.36 (1.69-3.28) | 2.19 (1.54-3.11) |
| Ophthalmic zoster | 1.93 (1.72-2.17) | 1.95 (1.73-2.19) |
| †Adjusted for age (modelled as a 5-knot restricted cubic spline to allow for non-linearity unless otherwise specified), gender, socioeconomic status, HIV, leukaemia, lymphoma, myeloma, hematopoietic stem cell transplantation, other unspecified cellular immune deficiencies, immunosuppressive therapies, rheumatoid arthritis, systemic lupus erythematosis, inflammatory bowel disease, COPD, asthma, chronic kidney disease, depression, personality disorder, diabetes, recent cancer diagnosis, smoking, BMI category, site of zoster and antiviral use. ¹ORs estimate the effect of a 10-year increase in age on PHN derived, in age groups <50, 50-79 and ≥80, from piecewise linear splines. ²Measured by Index of Multiple deprivation score. ³Includes patients currently taking a 14 day course of immunosuppressive medications, or terminating a 14 day course of immunosuppressive medications less than one month prior to the zoster diagnosis. Oral corticosteroid prescriptions were required to be high dose (≥20mg per day). | | |

| **Table e-8:** Associations between postherpetic neuralgia and demographic risk factors, comorbidities and health behaviours, stratified by whether a patient received antivirals during acute zoster. Analyses are restricted to 69,661 patients for whom antiviral status was most likely to be available* | **Not prescribed antivirals** | | | | **Prescribed antivirals** | | | |  |
| --- | --- | --- | --- | --- | --- | --- | --- | --- | --- |
|  | **Total cohort, n** | **Prevalence of PHN, n (%)** | **Age-adjusted OR (99% CI)** | **Fully adjusted† OR (99% CI)** | **Total cohort, n** | **Prevalence of PHN, n (%)** | **Age-adjusted OR (99% CI)** | **Fully adjusted† OR (99% CI)** | **P-value**†† |
| **Total** | 30302 (100) | 1416 (4.7) | - | - | 39359 (100) | 2250 (5.7) | - | - |  |
| ***Demographic characteristics*** |  |  |  |  |  |  |  |  |  |
| Age (years) [Estimated ORs for 10 year increase in age within the specified age-band]¹ | | | |  |  |  |  |  |  |
| Among those aged <50 | 9785 (32.3) | 152 (1.6) | 1.52 (1.26-1.83) | 1.43 (1.18-1.75) | 9738 (24.8) | 179 (1.8) | 1.32 (1.12-1.56) | 1.30 (1.08-1.55) | 0.401 |
| Among those aged 50-79 | 17273 (57.1) | 901 (5.2) | 1.71 (1.57-1.87) | 1.66 (1.52-1.82) | 24863 (63.3) | 1467 (5.9) | 1.77 (1.65-1.90) | 1.73 (1.60-1.86) | 0.465 |
| Among those aged ≥80 | 3200 (10.6) | 329 (10.3) | 1.05 (0.79-1.40) | 1.18 (0.86-1.64) | 4698 (12.0) | 563 (12.0) | 1.01 (0.80-1.28) | 1.05 (0.81-1.37) | 0.880 |
| Female | 17526 (57.8) | 919 (5.2) | 1.25 (1.07-1.45) | 1.24 (1.05-1.46) | 23851 (60.6) | 1453 (6.1) | 1.14 (1.01-1.28) | 1.14 (1.00-1.29) | 0.288 |
| Socioeconomic status (practice level)² |  |  |  |  |  |  |  |  |  |
| 1 (least deprived) | 5017 (16.6) | 205 (4.1) | 1.00 | 1.00 | 6773 (17.2) | 382 (5.6) | 1.00 | 1.00 | 0.636 |
| 2 | 6785 (22.4) | 291 (4.3) | 1.08 (0.83-1.42) | 1.07 (0.81-1.41) | 8996 (22.9) | 489 (5.4) |  | 0.97 (0.79-1.20) |  |
| 3 | 6301 (20.8) | 295 (4.7) | 1.12 (0.85-1.46) | 1.09 (0.82-1.44) | 8450 (21.5) | 477 (5.6) | 0.98 (0.79-1.21) | 0.98 (0.80-1.22) |  |
| 4 | 6595 (21.8) | 343 (5.2) | 1.29 (1.00-1.68) | 1.21 (0.93-1.59) | 8151 (20.7) | 513 (6.3) | 0.98 (0.79-1.21) | 1.10 (0.89-1.35) |  |
| 5 (most deprived) | 5604 (18.5) | 282 (5.0) | 1.32 (1.01-1.73) | 1.19 (0.90-1.58) | 6989 (17.8) | 389 (5.6) | 1.06 (0.85-1.32) | 1.02 (0.82-1.27) |  |
|  |  |  |  |  |  |  |  |  |  |
| ***Severe Immunosuppression*** |  |  |  |  |  |  |  |  |  |
| HIV | 22 (0.1) | 2 (9.1) | 4.35 (0.62-30.60) | 3.79 (0.47-30.34) | 45 (0.1) | 2 (4.4) | 1.71 (0.26-11.41) | 1.95 (0.29-13.09) | 0.540 |
| Leukaemia | 27 (0.1) | 7 (25.9) | 7.05 (2.13-23.32) | 7.12 (1.78-28.44) | 49 (0.1) | 4 (8.2) | 1.33 (0.34-5.21) | 1.39 (0.35-5.51) | 0.045 |
| Lymphoma | 60 (0.2) | 7 (11.7) | 2.81 (0.96-8.23) | 2.80 (0.87-9.04) | 110 (0.3) | 7 (6.4) | 1.16 (0.42-3.22) | 1.27 (0.45-3.56) | 0.199 |
| Myeloma | 63 (0.2) | 12 (19.1) | 3.31 (1.41-7.77) | 2.47 (0.94-6.45) | 124 (0.3) | 14 (11.3) | 1.59 (0.76-3.36) | 1.64 (0.77-3.48) | 0.250 |
|  |  |  |  |  |  |  |  |  |  |
| Oral corticosteroids³ | 87 (0.3) | 10 (11.5) | 2.31 (0.94-5.64) | 1.65 (0.60-4.49) | 137 (0.4) | 18 (13.1) | 2.12 (1.08-4.14) | 1.88 (0.94-3.77) | 0.792 |
| Other immunosuppressive therapy³ | 327 (1.1) | 25 (7.7) | 1.42 (0.82-2.46) | 1.09 (0.57-2.06) | 592 (1.5) | 42 (7.1) | 1.32 (0.87-2.02) | 1.05 (0.64-1.71) | 0.451 |
|  |  |  |  |  |  |  |  |  |  |
| ***Autoimmune conditions*** |  |  |  |  |  |  |  |  |  |
| Rheumatoid Arthritis | 495 (1.6) | 51 (10.3) | 1.72 (1.16-2.56) | 1.40 (0.86-2.26) | 911 (2.3) | 77 (8.5) | 1.26 (0.92-1.74) | 1.23 (0.86-1.75) | 0.112 |
| Systemic Lupus Erythematosis | 68 (0.2) | 10 (14.7) | 3.69 (1.47-9.31) | 3.42 (1.31-8.94) | 117 (0.3) | 7 (6.0) | 1.24 (0.45-3.45) | 1.11 (0.39-3.12) | 0.026 |
| Inflammatory Bowel Disease | 326 (1.1) | 24 (7.4) | 1.54 (0.88-2.70) | 1.53 (0.84-2.79) | 529 (1.3) | 39 (7.4) | 1.37 (0.88-2.13) | 1.22 (0.77-1.95) | 0.323 |
|  |  |  |  |  |  |  |  |  |  |
| ***Other comorbidities*** |  |  |  |  |  |  |  |  |  |
| COPD | 1092 (3.6) | 144 (13.1) | 2.01 (1.56-2.58) | 1.69 (1.29-2.22) | 1711 (4.4) | 202 (11.8) | 1.55 (1.26-1.90) | 1.42 (1.15-1.77) | 0.043 |
| Asthma | 1992 (6.6) | 106 (5.3) | 1.33 (1.01-1.75) | 1.38 (1.04-1.83) | 2839 (7.2) | 157 (5.5) | 1.12 (0.90-1.40) | 1.07 (0.84-1.34) | 0.088 |
| Chronic Kidney Disease | 1203 (4.0) | 116 (9.6) | 1.25 (0.96-1.65) | 1.14 (0.86-1.53) | 2173 (5.5) | 228 (10.5) | 1.26 (1.03-1.53) | 1.16 (0.95-1.42) | 0.916 |
| Depression | 1315 (4.3) | 70 (5.3) | 1.44 (1.03-2.00) | 1.21 (0.85-1.74) | 1797 (4.6) | 132 (7.4) | 1.62 (1.26-2.07) | 1.51 (1.17-1.95) | 0.475 |
| Personality disorder | 192 (0.6) | 13 (6.8) | 1.76 (0.82-3.76) | 1.64 (0.74-3.64) | 239 (0.6) | 13 (5.4) | 1.07 (0.51-2.26) | 0.93 (0.43-2.03) | 0.149 |
| Diabetes | 1924 (6.4) | 150 (7.8) | 1.24 (0.98-1.58) | 1.12 (0.87-1.44) | 2926 (7.4) | 265 (9.1) | 1.32 (1.10-1.58) | 1.26 (1.05-1.52) | 0.665 |
| Recent cancer diagnosis | 265 (0.9) | 20 (7.5) | 1.10 (0.60-2.04) | 1.17 (0.62-2.19) | 417 (1.1) | 37 (8.9) | 1.30 (0.83-2.05) | 1.42 (0.90-2.24) | 0.421 |
|  |  |  |  |  |  |  |  |  |  |
| ***Health behaviours and characteristics of zoster episode*** | | |  |  |  |  |  |  |  |
| Smoking |  |  |  |  |  |  |  |  |  |
| Non-smoker | 11583 (38.7) | 430 (3.7) | 1.00 | 1.00 | 14876 (38.0) | 791 (5.3) | 1.00 | 1.00 | 0.002 |
| Current smoker | 8505 (28.4) | 399 (4.7) | 1.66 (1.37-2.01) | 1.49 (1.21-1.83) | 9452 (24.2) | 469 (5.0) | 1.14 (0.97-1.33) | 1.09 (0.93-1.29) |  |
| Ex-smoker | 9857 (32.9) | 580 (5.9) | 1.38 (1.16-1.64) | 1.29 (1.07-1.56) | 14779 (37.8) | 981 (6.6) | 1.08 (0.95-1.23) | 1.03 (0.90-1.18) |  |
| BMI Category |  |  |  |  |  |  |  |  |  |
| Underweight (BMI <18.5) | 582 ( 2.1) | 39 (6.7) | 1.38 (0.87-2.17) | 1.18 (0.73-1.93) | 671 (1.9) | 65 (9.7) | 1.54 (1.07-2.20) | 1.45 (1.01-2.09) | 0.712 |
| Normal Weight (BMI 18.5-24.9) | 10981 (40.2) | 497 (4.5) | 1.00 | 1.00 | 14125 (39.2) | 787 (5.6) | 1.00 | 1.00 |  |
| Overweight (BMI 25-30) | 9942 (36.4) | 473 (4.8) | 0.96 (0.81-1.14) | 1.01 (0.84-1.21) | 13320 (37.0) | 793 ( 6.0) | 1.03 (0.90-1.18) | 1.04 (0.90-1.19) |  |
| Obese (BMI ≥30) | 5818 (21.3) | 317 (5.5) | 1.21 (1.00-1.47) | 1.15 (0.94-1.42) | 7921 (22.0) | 486 (6.1) | 1.17 (1.00-1.37) | 1.12 (0.95-1.32) |  |
| †Adjusted for age, gender, socioeconomic status, HIV, leukaemia, lymphoma, myeloma, other unspecified cellular immune deficiencies, immunosuppressive therapies, rheumatoid arthritis, systemic lupus erythematosis, inflammatory bowel disease, COPD, asthma, chronic kidney disease, depression, personality disorder, diabetes, recent cancer diagnosis, smoking and BMI category. Please note hematopoietic stem cell transplantation and other unspecified cellular immune deficiencies were excluded due to too few numbers. Interaction terms between antiviral use and other risk factors were added to the model one at a time. ††P-value for interaction. ¹ORs estimate the effect of a 10-year increase in age on PHN derived, in age groups <50, 50-79 and ≥80, from piecewise linear splines. ²Measured by Index of Multiple deprivation score. ³Includes patients currently taking a 14 day course of immunosuppressive medications, or terminating a 14 day course of immunosuppressive medications less than one month prior to the zoster diagnosis. Oral corticosteroid prescriptions were required to be high dose (≥20mg per day). *Patients excluded were those diagnosed with zoster in HES, or with a hospital visit for zoster (primary diagnosis of any episode) in the 7 days after first zoster diagnosis, patients without linked HES data and patients with evidence of non-truncal zoster, as their antiviral use may not be recorded in CPRD. | | | | | | | | | |

**References**

1. Centers for Disease Control and Prevention, *Prevention of Herpes Zoster: Recommendations of the Advisory Committee on Immunization Practices (ACIP)*. 2008.

2. Rait G, Walters K, Griffin M, et al. Recent trends in the incidence of recorded depression in primary care. Br J Psychiatry 2009: 195(6): 520-4.

3. Royal College of General Practitioners, *Coding, Classification and Diagnosis of Diabetes*, NHS, Editor. 2011.

4. Mulnier HE, Seaman HE, Raleigh VS, et al. Mortality in people with Type 2 diabetes in the UK. Diabetic Medicine 2006: 23(5): 516-521.

5. Andersohn F, Schade R, Suissa S, and Garbe E. Long-Term Use of Antidepressants for Depressive Disorders and the Risk of Diabetes Mellitus. American Journal of Psychiatry 2009: 166(5): 591-598.

6. Langan SM, Minassian C, Smeeth L, and Thomas SL. Risk of stroke following herpes zoster: a self-controlled case-series study. Clin Infect Dis 2014: 58(11): 1497-503.

7. Rubben A, Baron JM, and GrussendorfConen EI. Routine detection of herpes simplex virus and varicella zoster virus by polymerase chain reaction reveals that initial herpes zoster is frequently misdiagnosed as herpes simplex. British Journal of Dermatology 1997: 137(2): 259-261.

8. Parruti G, Tontodonati M, Rebuzzi C, et al. Predictors of pain intensity and persistence in a prospective Italian cohort of patients with herpes zoster: relevance of smoking, trauma and antiviral therapy. BMC Medicine 2010: 8: 58.

9. Drolet M, Brisson M, Schmader K, et al. Predictors of postherpetic neuralgia among patients with herpes zoster: a prospective study. The journal of pain: official journal of the American Pain Society 2010: 11(11): 1211-21.

10. Steenland K. Smoothing is soothing, and splines are fine. Occupational and Environmental Medicine 2005: 62(3): 141-142.

11. White IR, Royston P, and Wood AM. Multiple imputation using chained equations: Issues and guidance for practice. Statistics in medicine 2011: 30(4): 377-99.

12. Chen N, Li Q, Yang J, et al. Antiviral treatment for preventing postherpetic neuralgia. Cochrane Database Syst Rev 2014: 2: CD006866.
